# Replicability of Functional Brain Networks: A Study Through the Lens of Seven Resting‐State Networks

**DOI:** 10.1002/hbm.70559

**Published:** 2026-06-08

**Authors:** Kaitlyn R. Fales, Xurui Zhi, Hyebin Song, Nicole A. Lazar

**Affiliations:** ^1^ Department of Statistics Pennsylvania State University University Park Pennsylvania USA; ^2^ Huck Institutes of the Life Sciences Pennsylvania State University University Park Pennsylvania USA

**Keywords:** band‐pass filtering, brain parcellation, data preprocessing, functional connectivity, functional network, replicability, resting‐state fMRI

## Abstract

The study of brain networks is essential for improving our understanding of how the human brain functions. Functional connectivity (FC) analysis is a widely used approach for studying co‐activating patterns among brain regions by estimating their temporal dependencies and constructing an undirected network. Data processing is critical before estimating a subject's functional network, but the absence of a standardized procedure serves as a source of heterogeneity in results, especially in multi‐site studies. Commonly studied functional networks include the default mode, sensorimotor, visual, salience, dorsal attention, frontoparietal, and language networks. These networks are stable and still exhibit intrinsic activation when an individual is at rest, making them ideal networks to focus on for studying how processing choices affect the replicability of functional connectivity networks. We use the aforementioned seven networks to assess the impact of various processing choices, including preprocessing pipeline, band‐pass filtering, and brain parcellation, on the replicability of functional connectivity estimates for multi‐site resting‐state fMRI (rs‐fMRI) data from the Autism Brain Imaging Data Exchange (ABIDE). Finally, we provide some practical recommendations for how researchers should proceed with processing choices in the face of these effects.

## Summary

- This study examines the effects of preprocessing pipelines, 0.01–0.1 Hz band‐pass filtering, and brain parcellation on the replicability of functional connectivity (FC) estimates; we find that pipeline and parcellation, as well as their interaction, significantly impact FC estimates, while band‐pass filtering appears to have a limited effect.
- A key contribution of our work is the comprehensive examination of all three factors in assessing their impact on FC estimation, utilizing a large sample in a multi‐site setting.
- These findings highlight the effects of different processing choices on FC estimates, which emphasize the importance of full disclosure, justification, and discussion of methods used.

## Graphical abstract

This study assesses the impact of preprocessing pipeline, 0.01–0.1 Hz band‐pass filtering, and brain parcellation on the replicability of functional connectivity estimates in seven resting‐state networks for multi‐site fMRI data. We find large effects for pipeline and parcellation on FC estimates, while band‐pass filtering has little effect.

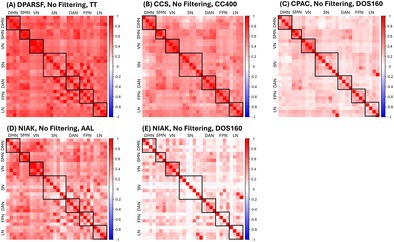

## 1 Introduction

Reproducible and replicable findings are crucial for scientific advancement in all fields, and neuroimaging is no exception. The results of a study are considered “reproducible” if a research team achieves the same results as another team using identical data and methods; results are considered “replicable” if consistent results are obtained across studies that address the same research question even if the data and methods vary (National Academies of Sciences, Engineering, and Medicine et al. 2019). There is growing interest in open science practices that can lead to greater reproducibility and replicability, but adoption of these practices has been slow, due to barriers such as fears and policies that prevent or limit data sharing, lack of experience with resources for the setup and maintenance of data and code repositories, and complexity of research (Niso et al. 2022; Paret et al. 2022).

For functional neuroimaging techniques such as functional MRI (fMRI), there are additional barriers that hinder the replicability of results. In particular, raw fMRI data from the MR scanner must undergo multiple preprocessing steps before statistical analysis (Ashby 2015; Weber et al. 2015). While there is general agreement on a broad set of preprocessing steps required for fMRI analysis, such as correcting slice timing differences, standardizing spatial location of images, and removing outliers from the data, the flexibility in selecting methods and ordering steps still leads to a combinatorial explosion of possible workflows (Carp 2012; Luppi et al. 2024). The flexibility in data analysis configurations is referred to as “researcher degrees of freedom” (Simmons et al. 2011) in psychology, and we argue that these degrees of freedom hinder replicability not just in terms of data analysis, but also data preprocessing. The flexibility in preprocessing choices, when considering fMRI data specifically, impacts study results as well as reproducibility and replicability of findings (Poldrack et al. 2017).

Replicability concerns in fMRI are further exacerbated by the issue of low statistical power in many studies. Low statistical power is a consequence of small sample sizes, with the median estimated sample size for a single‐group fMRI study in 2015 being 28.5, and for a multi‐group study being 19 subjects per group (Poldrack et al. 2017). Acquiring a large sample from a single fMRI experiment is difficult, so multi‐site studies are growing in popularity (Bari et al. 2019; Deprez et al. 2018; Yan et al. 2013; Yu et al. 2018), especially when the research involves studying health conditions such as Alzheimer's disease or autism (Abraham et al. 2017). However, while multi‐site studies increase sample sizes, they also introduce additional sources of variation that make statistical analysis more complex. Even when MR scanners from each site have strict quality control procedures and scanning parameters across scanners and sites are harmonized, site variability is still present and can detract from the sample size advantage of multi‐site studies (Bari et al. 2019; Deprez et al. 2018).

One area of neuroimaging research affected by these reproducibility challenges is functional connectivity (FC) analysis. FC is a popular neuroimaging research area which investigates temporal dependencies between anatomically distinct brain regions through measures of correlation or coherence (Büchel and Friston 1997; Friston 1994; Hlinka et al. 2011; Mohanty et al. 2020; Raichle 2011; Shahhosseini and Miranda 2022; Smith et al. 2013). In this paper, we focus on three key processing factors that can influence the reproducibility of FC findings: (1) preprocessing pipeline, (2) temporal filtering, and (3) brain parcellation.

Preprocessing pipeline choices have been shown to impact downstream fMRI analyses, whether for functional connectivity or task‐based activation (Botvinik‐Nezer et al. 2020; Carp 2012; Luppi et al. 2024; Shirer et al. 2015). Here, a preprocessing pipeline refers to the specific set and sequence of preprocessing steps. In the study by Carp (2012), 6912 unique analysis pipelines are compared for task‐based activation patterns. The 6912 pipelines are from all possible combinations of four preprocessing steps and six analysis steps, each of which has a number of options. It is found that all four preprocessing steps—despiking, slice timing correction, normalization, and spatial smoothing—contribute to variation in task activation analysis. In contrast, Botvinik‐Nezer et al. (2020) find that differences in preprocessing choices for task‐based analysis are less important compared to choices for the subsequent statistical analysis when it comes to variability in results. Within FC specifically, Luppi et al. (2024) conduct an analysis of 768 pipelines for functional network reconstruction, evaluating the effects of brain parcellation, definition of connectivity, and global signal regression (GSR); they find systematic variability in network reconstruction, with 38 pipelines being misleading in the sense that effects do not replicate in terms of magnitude or even direction, preventing overall network replication. Even when combining individual analysis steps that seem reasonable, the end‐to‐end pipeline is hindering FC analysis and replicability of results (Luppi et al. 2024; Shirer et al. 2015).

To help address this problem and promote standardization within the field, it is worth mentioning fMRIPrep (Esteban et al. 2019), which has quickly become one of the most widely‐used preprocessing tools. With version 1.0 first available for use in 2018, fMRIPrep is a preprocessing tool that can be used regardless of the choice of downstream statistical analysis (i.e., task‐based activation, resting‐state functional connectivity, etc.); fMRIPrep combines robust workflows from different neuroimaging softwares (e.g., FSL, AFNI, etc.) into a single, unified software. The use of fMRIPrep helps standardize preprocessing pipelines by reducing manual user intervention, producing verbose quality control outputs, and promoting ease of use through the Brain Imaging Data Structure (BIDS) standards (Esteban et al. 2019; Gorgolewski et al. 2017). Even with access to this tool, researcher degrees of freedom are still present, as users can choose some parameters throughout the pipeline. There are also a variety of other available softwares and workflows which labs can choose from to conduct their data preprocessing. There is no pipeline that is universally superior for preprocessing fMRI data, but using standardized preprocessing workflows may improve replicability by reducing differences that arise purely from subjective or laboratory‐specific choices.

Two other key processing steps performed after the pipeline but prior to FC analysis are temporal filtering and brain parcellation. The purpose of temporal filtering is to filter out noise from frequencies not contributing to FC activation and preserve the signal from frequencies that contribute to activation (Cordes et al. 2001; Sala‐Llonch et al. 2019; Shirer et al. 2015). Low frequencies, or frequencies below 0.1 Hz, are where most of the signal in fMRI is concentrated (Cordes et al. 2001), and as such, most types of temporal filtering involve filtering out higher frequencies. Shirer et al. (2015) investigate different band‐pass filter choices for FC analysis and find that higher frequency cutoffs, namely a band‐pass filter of 0.01–0.1 Hz or a high‐pass filter of 0.01 Hz, improve signal‐noise separation and test–retest reliability more than 0.01–0.03 and 0.03–0.07 Hz filters. Sala‐Llonch et al. (2019) find that the effect of filtering depends on brain parcellation and connectivity measure choice. When using Pearson correlation as the connectivity measure, parcellations—both prespecified and data‐driven—perform similarly when using no temporal filtering versus a lower frequency 0.005–0.096 Hz band‐pass filter (Sala‐Llonch et al. 2019). In the current study, we investigate the impact of a 0.01–0.1 Hz band‐pass filter versus no filter on FC network replicability.

For FC analysis, the choice of brain parcellation to form the regions‐of‐interest (ROIs) is both important and difficult because there are many prespecified atlases in circulation, along with data‐driven procedures, leaving no formal consensus on the functional organization of the brain (Bijsterbosch et al. 2020; Eickhoff et al. 2018; Messé 2020; Papo et al. 2014). As for the effect of the parcellation itself on FC analysis, Luppi et al. (2024) find that parcellation choice is important, but only within the context of the entire pipeline, so the pipeline should inform parcellation choice. Other literature shows that parcellation choice has a significant effect on FC, but mostly from the number of parcels or granularity of the atlas as opposed to the method itself (Arslan et al. 2018; Messé 2020).

In this study, we systematically assess how different data processing choices impact the replicability of functional connectivity networks. Specifically, we examine the effects of preprocessing pipelines, temporal filtering, and brain parcellation, while accounting for the added complexity of site‐to‐site variability due to the use of multi‐site fMRI data. Here, we note that while we refer to the three effects of interest broadly as “processing choices”, there is an important distinction between them. Pipeline and band‐pass filtering are strictly preprocessing steps for the purpose of data cleaning, whereas choice of parcellation is a modeling choice that reflects assumptions about the organization of the brain. As all three steps occur prior to the creation of the FC networks, we refer to the collection of these effects as data processing choices only for ease of explanation.

We focus on seven commonly studied functional networks: the default mode (DMN), sensorimotor (SMN), visual (VN), salience (SN), dorsal attention (DAN), frontoparietal (FPN), and language (LN) networks. Intrinsic brain activity involves the same functional networks regardless of if the subject is completing a task (Raichle 2011; Seitzman et al. 2019; Smith et al. 2013). As such, we can study the existing networks between functionally connected brain regions when the subject is at rest, motivating the use of rs‐fMRI as the primary vehicle for studying the connectome, or the comprehensive brain network (Smith et al. 2013).

All of these networks, with the exception of the default mode, are “task positive networks”, meaning they are most active when a subject is actively engaged in a task (Seitzman et al. 2019). The DMN, however, is a network that “runs in the background”, meaning it is most active when a subject is unfocused or at rest (Buckner et al. 2008; McCormick and Telzer 2018; Seitzman et al. 2019). These seven networks are well studied and generally considered stable across subjects even with individual subject‐level variability (Goelman et al. 2024; Gratton et al. 2018; Raichle 2011), making them well suited for studying how data processing choices affect the replicability of functional connectivity estimates. While prior studies have shown that preprocessing pipeline, temporal filtering, and parcellation all affect functional connectivity networks, most studies have focused on only one or two of these aspects, and/or studied using limited sample sizes. In contrast, we provide a comprehensive evaluation of their individual and interaction effects using a large, multi‐site dataset.

## 2 Materials and Methods

### 2.1 ABIDE I Dataset

We use data from the Autism Brain Imaging Data Exchange (ABIDE) (Craddock et al. 2013; Di Martino et al. 2014). The ABIDE I dataset is a collection of resting‐state fMRI (rs‐fMRI) data from 17 international neuroimaging sites including 539 individuals with autism spectrum disorder (ASD) and 573 controls. Each neuroimaging site uses a model from one of three scanner brands—Siemens, Phillips, and GE—and sets its own scanning parameters (repetition time, echo time, flip angle, field‐of‐view, etc.). Additionally, each site provides the ABIDE developers with the raw images, allowing them to preprocess the data before making it publicly available.

Since there is no universally agreed upon pipeline for processing rs‐fMRI data, the ABIDE developers provide a variety of processed versions, including four pipelines, options for band‐pass filtering and GSR, and seven brain parcellations. The levels of each factor are given in Table 1. At the time of the ABIDE I dataset being made available, a standardized tool such as fMRIPrep did not yet exist. The four preprocessing pipelines used by the ABIDE developers were some of the best available options at the time. Therefore, we work with the processed data made available from the ABIDE developers, using only the pipelines they chose. This study design choice helps enforce fairness in the comparisons we wish to make. The goal of the current study is not to find a single “best” pipeline, but rather to understand the variability that can arise from different choices within a shared framework. The shared framework here is the combinations of processed ABIDE data made publicly available for researchers to use.

**TABLE 1:** Combinations of processing choices from the ABIDE I dataset.

Effect	Configurations
Preprocessing pipeline	Connectome Computation System (CCS) Configurable Pipeline for the Analysis of Connectomes (CPAC) Data Processing Assistant for Resting‐State fMRI (DPARSF) Neuroimaging Analysis Kit (NIAK)
Band‐pass filtering	0.01–0.1 Hz band‐pass filter applied No filtering applied
Global signal regression	Global signal regression applied No global signal regression applied
Brain parcellation	Automated Anatomical Labeling (AAL) Craddock 200 (CC200) Craddock 400 (CC400) Dosenbach 160 (DOS160) Eickhoff‐Zilles (EZ) Harvard‐Oxford (HO) Talaraich and Tournoux (TT)

For every subject and combination of pipeline, filtering, and GSR, there are data files containing the mean BOLD time series across the voxels in every parcel according to each of the seven brain parcellations. We use these processed data to conduct the analysis, with the exception of the samples processed using GSR. We do not use samples with GSR given the controversies surrounding the method, including the introduction of spurious negative correlations (Murphy et al. 2009) and its lack of effectiveness as a data standardization method in reducing inter‐subject and inter‐site variability (Yan et al. 2013). Furthermore, there is additional heterogeneity in functional networks for individuals with ASD (Di Martino et al. 2014), so we restrict the sample to only include the neurotypical control subjects.

It is well‐known that differences in handedness affect functional connectivity estimates (Pool et al. 2015; Tejavibulya et al. 2022, 2025; Tomasi and Volkow 2024), so we only use data from right‐handed subjects. To address quality control, we leverage the automated and manual quality assessment (QA) protocol provided by the ABIDE developers. Using this, we restrict our sample to subjects with a mean framewise displacement (FD) less than or equal to 0.2 mm and a percentage of volumes with FD greater than 0.2 mm less than or equal to 25 percent to control for head motion effects. Any subjects whose data fail the manual inspection of anatomical or functional images by independent raters are also excluded. More details regarding our use of ABIDE's QA protocol for the quality control in the current study can be found in Supporting information S1.

After implementing the inclusion criteria, the current study has a sample size of n=282, but some processing combinations contain data from as few as 268 subjects, as some subjects have missing values for ROIs where the BOLD signals contain only zeroes, making the dataset slightly imbalanced across combinations. The sample mean age is about 16 years with a standard deviation of 6.49 years. Our areas of interest are the choice of preprocessing pipeline, band‐pass filtering, and brain parcellation on FC variability in seven common resting‐state networks both within and between subjects. We investigate these impacts using all four pipelines, the choice of filtering or not, and all seven atlases, giving 56 possible processing combinations.

#### 2.1.1 Preprocessing Pipelines

The ABIDE developers implement four common pipeline choices for rs‐fMRI data, as shown in Table 1. For each pipeline, the developers use the default settings and parameter values from the pipeline developers (ABIDE 2013). In general, the four pipelines follow the same preprocessing steps, but they differ in terms of algorithm, software, and/or parameters used within steps.

The Connectome Computation System (CCS) (Xing et al. 2022; Xu et al. 2015) uses functions from AFNI (Cox 1996), FSL (Jenkinson et al. 2012), FreeSurfer (Fischl 2012), and SPM (RRID:SCR_007037) for preprocessing. The Configurable Pipeline for the Analysis of Connectomes (CPAC) is an open‐source software tool using Python that integrates functions from AFNI, FSL, and Python. The Data Processing Assistant for Resting‐State fMRI (DPARSF) is a plug‐in software based on SPM and REST (Song et al. 2011). Finally, the Neuroimaging Analysis Kit (NIAK) is an open‐source software compatible with Octave and Matlab. Though the NIAK pipeline is no longer maintained as of 2020 (NITRC 2024), we include it in the comparison because it has been a common choice for preprocessing rs‐fMRI data (Orban et al. 2015; Therriault et al. 2019). As for the other pipelines, DPARSF has been merged into the Data Processing and Analysis for Brain Imaging (DPABI) toolbox (Yan et al. 2016) and is still in use for resting‐state data, as are CCS and CPAC, but all three are less popular tools for preprocessing in comparison to fMRIPrep.

The key differences among the four pipelines are as follows, with Table 2 summarizing these differences. CCS and DPARSF drop the first four brain volumes; the others do not drop any. NIAK is the only pipeline that does not perform slice timing correction. For intensity normalization, CCS and CPAC both use a 4D global mean procedure, NIAK performs a correction using the median volume, and DPARSF does not use any form of correction. All pipelines conduct some form of nuisance signal regression, but for head motion and low‐frequency drifts, NIAK differs from the other three, and for tissue signals (e.g., WM, CSF), CPAC differs from the other three.

**TABLE 2:** Summary of key preprocessing differences across ABIDE pipelines.

Step	CCS	CPAC	DPARSF	NIAK
Volumes removed	1: First four		1: First four	
Slice timing correction	2: AFNI (3dTshift)	1: AFNI (3dTshift)	2: SPM (Slice Timing)	
Motion correction	3: AFNI (3dvolreg)	2: AFNI (3dvolreg)	3: SPM (Realign)	2: Rigid body
Intensity normalization	4: 4D global mean	3: 4D global mean		1: N3, median volume
Co‐registration	5: Boundary‐based (FreeSurfer bbregister)	6: Boundary‐based (FSL FLIRT)	4: Rigid body (SPM Coregister)	3: Rigid body
Nuisance regression				
Motion	6: 24‐param motion Mean WM and CSF Linear and quadratic	4: 24‐param motion WM and CSF (5 PCs) Linear and quadratic	5: 24‐param motion Mean WM and CSF Linear and quadratic	5: Motion scrubbing, Six motion (1 PC) Mean WM and CSF Discrete cosine basis
Tissue				
Drift				
Band‐pass filtering (if applicable)	7: 0.01–0.1 Hz	5: 0.01–0.1 Hz	6: 0.01–0.1 Hz	6: 0.01–0.1 Hz
Normalization (MNI152)	8: FLIRT, FNIRT (FSL)	7: ANTs	7: DARTEL	4: CIVET
Spatial smoothing			8: FWHM 6 mm	7: FWHM 6 mm

*Note:* Rows are ordered according to the CCS pipeline procedure, and numbers for each pipeline indicate where the ordering of steps deviates from CCS. Entries highlight primary software tools and parameter choices where pipelines differ to the best of our knowledge based on ABIDE (2013) documentation; blank entries indicate the steps the various pipelines do not perform. Full details are provided in Supporting information S2.

For the transformation from native to template (MNI152) space, the procedures all vary, but do involve a combination of functional to anatomical and anatomical to template transformations. For the functional to anatomical transformations, CCS and CPAC use a boundary‐based rigid body procedure, and DPARSF and NIAK use a rigid body procedure. The anatomical to template transformations all differ, with CCS using FLIRT and FNIRT in FSL, CPAC using ANTs (Tustison et al. 2021), DPARSF using DARTEL (Ashburner 2007), and NIAK using CIVET (Zijdenbos et al. 2002). For more information on the specifications of each pipeline using the appropriate version at that time, we refer the reader to Supporting information S2, as well as the complete descriptions on the ABIDE website (http://preprocessed‐connectomes‐project.org/abide/Pipelines.html).

#### 2.1.2 Band‐Pass Filtering

Another preprocessing step includes 0.01–0.1 Hz band‐pass filtering, which occurs in the frequency domain, and removes signals occurring at frequencies less than 0.01 Hz and greater than 0.1 Hz. We consider the effect of band‐pass filtering versus no filtering on FC estimation. For the ABIDE dataset, we observe that, for the NIAK pipeline, the preprocessed BOLD data are identical between the filtered and unfiltered versions for all subjects. We reached out to the ABIDE developers regarding this observation but did not receive a response. Inspection of the power spectra of representative time series is consistent with band‐pass filtering in the CCS, CPAC, and DPARSF pipelines, but not in NIAK, indicating that band‐pass filtering is not applied in the NIAK pipeline.

#### 2.1.3 Brain Parcellation

After preprocessing the data according to the eight pipeline/filtering combinations, the mean BOLD signal is extracted for every brain region defined according to seven different atlases. The seven atlases are given in Table 1. The number of parcels in the seven parcellations ranges from 110 (HO and TT) to 400 (CC400). Also, methods for constructing the atlases differ from one another, and there are some inconsistencies in the anatomical labels between atlases. For example, two overlapping parcels in the AAL versus TT atlases are defined as “Frontal Medial Orbital” and “Medial Frontal Gyrus”, respectively. To facilitate direct comparison of functional connectivity across atlases with differing spatial resolutions and labeling conventions, we impose a common network‐level framework by mapping each parcellation to a fixed set of reference ROIs. This allows us to isolate the effect of parcellation choice on downstream connectivity estimates while avoiding subjective region selection within each atlas.

For our purposes, we use the ROI definitions from the CONN toolbox networks atlas (Nieto‐Castanon and Whitfield‐Gabrieli 2020). The CONN toolbox networks atlas contains volume information for the seven networks of interest, plus two ROIs in the cerebellar network, with 32 total regions from a sample of 497 healthy controls. The seven networks of interest, along with the ROI names, and MNI coordinates used in the CONN atlas are shown in Table 3. We note that large‐scale functional network definitions, including the seven‐network organization from CONN, are not uniquely determined and may vary depending on the methodologies used to determine the networks. In the current study, we adopt the CONN networks framework as a standardized reference to enable consistent comparisons across different processing choices. Our procedure for determining which parcels from each of the seven parcellations constitute the ROIs for each network is based on the proportion of overlap with the CONN network ROIs.

**TABLE 3:** Regions included in the CONN toolbox networks atlas.

Network	Region	*x*	*y*	*z*	Network	Region	*x*	*y*	*z*
Default mode	MPFC	1	55	−3	Salience	ACC	0	22	35
	LP_L	−39	−77	33		AInsula_L	−44	13	1
	LP_R	47	−67	29		AInsula_R	47	14	0
	PCC	1	−61	38		RPFC_L	−32	45	27
Sensorimotor	Lateral_L	−55	−12	29		RPFC_R	32	46	27
	Lateral_R	56	−10	29		SMG_L	−60	−39	31
	Superior	0	−31	67		SMG_R	62	−35	32
Visual	Medial	2	−79	12	Frontoparietal	LPFC_L	−43	33	28
	Occipital	0	−93	−4		PPC_L	−46	−58	49
	Lateral_L	−37	−79	10		LPFC_R	41	38	30
	Lateral_R	38	−72	13		PPC_R	52	−52	45
Dorsal attention	FEF_L	−27	−9	64	Language	IFG_L	−51	26	2
	FEF_R	30	−6	64		IFG_R	54	28	1
	IPS_L	−39	−43	52		pSTG_L	−57	−47	15
	IPS_R	39	−42	54		pSTG_R	59	−42	13

*Note:* The regions included in the CONN toolbox networks atlas (Nieto‐Castanon and Whitfield‐Gabrieli 2020) for the seven networks of interest. The MNI coordinates for the center of each region are also included.

We reslice the CONN atlas from 2 to 3 mm^3^ space for overlap comparison with each of the seven atlases using fourth degree B‐spline interpolation in SPM12 to ensure they are in the same space. All parcels in each atlas that have any amount of overlap with the corresponding CONN region are included in that region, but the parcel weights are determined by the proportion of the CONN region that the parcels cover. As an example, for the AAL atlas, we wish to determine which parcels form the MPFC region in the DMN. We identify all parcels within the AAL atlas that have any spatial overlap with the CONN networks MPFC region. In this case, there are eight such regions. The regions are then weighted based on the relative proportion of the CONN MPFC they cover. For the MPFC region using the AAL atlas, the two parcels with the largest proportions are “Frontal_Med_Orb_L” and “Frontal_Med_Orb_R”, with weights 0.304 and 0.251, respectively.

This procedure is repeated for all networks and all atlases to identify the parcels we include, as well as their corresponding weights, when forming the functional networks in Section 2.2. For all atlases and networks, the parcel indices, labels (if applicable), and weights are available on the study's GitHub repository. For some regions close to the outer boundaries of the brain, occasionally a small proportion of the CONN region is attributed to no region, or outside of the brain. In these cases, a reweighting is performed to ensure that CONN voxels deemed outside the brain (or not part of any specified region) by that atlas are not included, and the weights will still sum to one.

Unlike the other six atlases, the Dosenbach 160 (DOS160) atlas does not partition the entire brain. Instead, the atlas only specifies 4.5 mm radius spheres around the center of each region (ABIDE 2013). This presented a slight challenge when identifying which parcels to include, as there were two regions in which no parcel interacted with the CONN region: “SMG_R” in the salience network, and “FEF_R” in the dorsal attention network. In order to have complete data to conduct the analysis, we compute the distance between the centroid of every DOS160 parcel and the MNI centroid for each of the two affected regions from the CONN atlas (given in Table 3), using three distinct distance measures. We calculate the Euclidean, Manhattan, and Minkowski (with p=5) distances, identify the DOS160 parcel that is the closest to the CONN region for each measure, and then choose the parcel that is closest by majority vote. The chosen parcel for each region is assigned to its corresponding region with a weight of one. This additional procedure was only necessary for the two regions mentioned above; the other 28 regions proceeded in the same way as for the other atlases.

We recognize that the mapping strategy we use differs from more common approaches in which voxel‐level time series are directly summarized within predefined ROIs. However, a goal of the study is to assess how different parcellation schemes, provided as part of the ABIDE preprocessing outputs, affect downstream functional connectivity when a consistent network‐level framework is imposed. Because the atlases differ substantially in spatial resolution, labeling conventions, and anatomical boundaries, directly defining networks within each atlas requires subjective region selection and does not permit a standardized comparison. By fixing the network‐level ROIs using the CONN atlas and mapping each parcellation to these ROIs via spatial overlap weighting, we aim to isolate the effect of parcellation choice on network‐level connectivity estimates. This allows for a consistent comparison across heterogeneous atlases and enables the assessment of how differences in parcel definitions and spatial boundaries emerge in the resulting network‐level time series and connectivity estimates.

### 2.2 Functional Connectivity Network Estimation

To construct the functional networks, we first find the mean BOLD time series for each of the 30 total regions across the seven networks of interest. For any particular atlas, we use the proportions derived in Section 2.1.3 to take a weighted average of the signals across the regions to end with one BOLD signal per ROI.

For each pipeline j, band‐pass filter k, and atlas ℓ, let $c=\left(j,k,\ell \right)$ represent one of the 56 processing combinations. Let $x_{t,p}^{\left(i,c\right)}$ denote the BOLD signal at time t, in parcel p (within atlas ℓ), for the ith subject and combination c. Then, we find the mean BOLD time series $y_{t,s}^{\left(i,c\right)}$ for ROI s by, $y_{t,s}^{\left(i,c\right)}=\sum_{p\in R\left(s\right)}x_{t,p}^{\left(i,c\right)}w_{p}^{\left(c\right)}$, where $R\left(s\right)=\left\{p;p\;\text{is part of}\;\mathrm{ROI}\;s\right\}$ and $w_{p}$ is the corresponding weight for parcel p. We complete this process for all 56 processing combinations for every subject, resulting in 56 dataframes of dimension $T_{i}\times 30$, where $T_{i}$ refers to the number of scans (volumes) collected in the scanning session for subject i. We construct the FC network for every combination c and every subject i by calculating all pairwise Pearson correlations between the 30 ROIs, giving a 30×30 FC matrix, or a weighted network with 30 nodes and 435 edges. More specifically, for each pair $\left(i,c\right)$, $r_{e}^{\left(i,c\right)}=\text{Corr}\left(y_{s_{1}}^{\left(i,c\right)},y_{s_{2}}^{\left(i,c\right)}\right)$, where e is the edge between ROIs $s_{1}$ and $s_{2}$.

Additionally, to assess the potential impact of acquisition‐related transient effects in the initial volumes of the BOLD time series, which are handled differently across preprocessing pipelines (Table 2), we conduct a brief sensitivity analysis examining the influence of initial volume inclusion on functional connectivity estimates. For the pipelines that do not remove initial volumes (CPAC and NIAK), we recompute functional connectivity for all subjects using the AAL atlas after removing the first four volumes and compare the resulting connectivity matrices to the original estimates. Connectivity patterns are consistent, with correlations between vectorized matrices exceeding 0.94 in all cases, indicating minimal impact on downstream analyses.

### 2.3 Edgewise Modeling of Heterogeneity Effects

We model the connectivity of each edge separately using linear mixed effects models (LMMs). For the response variable, we use the Fisher's z‐transformed correlation coefficients. For a correlation coefficient r, Fisher's z‐transformation of r to an approximately normally distributed z is $z=\left(1/2\right)\left[\ln\left(1+r\right)-\ln\left(1-r\right)\right]=\text{arctanh}\left(r\right)$, where $\text{arctanh}\left(\cdot \right)$ is the inverse hyperbolic tangent. We then use LMMs to examine the effects of interest on the estimated connectivity of the FC network both within each network and between networks, while accounting for the repeated measures across the 56 combinations for each subject and variations across sites.

While there are 16 unique neuroimaging sites (as we discard the one remaining sample from CMU after implementing the sample inclusion criteria), four sites include multiple waves of independently collected samples: Ludwig Maximilians University Munich (MaxMun), University of California Los Angeles, University of Leuven, and the University of Michigan. Here, “waves” refer to distinct cohorts contributed separately by the same site, reflecting independent data collection efforts and non‐overlapping participants. For the MaxMun data, the number of subjects is small in each wave, with seven in the first wave, fourteen in the second, and three in the third. We follow the approach of the ABIDE developers and combine the subjects across the waves for this site to ensure adequate sample size. All other sites and waves have at least nine subjects and are therefore treated as separate site‐level samples to preserve potential between‐sample heterogeneity, resulting in a total of 19 sites.

For each edge $e\in \left\{1,\ldots ,435\right\}$, we consider two linear mixed effects models: one that models the additive fixed effects of preprocessing pipelines, band‐pass filtering, and atlas choice, together with subject‐specific random effects, and another that includes all additive fixed effects and their interactions, together with site‐specific random effects and subject‐specific random effects nested within sites. We use the first model (additive effects model) for each edge as an exploratory step to check main effects of the factors of interest before investigating higher‐order effects. The second models the full effects, including interactions.

The first model is given by, for each subject i and edge e,

$$z_{\mathrm{ijk}\ell }^{\left(e\right)}=\left(\mu^{\left(e\right)}+\eta_{i}^{\left(e\right)}\right)+\alpha_{j}^{\left(e\right)}+\beta_{k}^{\left(e\right)}+\gamma_{\ell }^{\left(e\right)}+\epsilon_{\mathrm{ijk}\ell }^{\left(e\right)}$$

where $\alpha_{j}$, $\beta_{k}$, $\gamma_{\ell }$ represent the fixed effects of pipeline j=1,…,4, filtering k=1,2, and atlas ℓ=1,…,7, $\eta_{i}^{\left(e\right)}∼N\left(0,{\left(\sigma_{\eta }^{\left(e\right)}\right)}^{2}\right)$ are subject‐specific random effects, which are independent across subjects i and network edges e, and $\epsilon_{\text{ijk}\ell }^{\left(e\right)}\overset{\text{iid}}{∼}N\left(0,{\left(\sigma^{\left(e\right)}\right)}^{2}\right)$ are the errors. Note for identifiability, we assume the sum across all levels of each fixed effect is zero (e.g., for pipeline, $\sum_{j=1}^{4}\alpha_{j}^{\left(e\right)}=0$). We summarize the notation for the model fixed effects in Table 4. The results of the model fits suggest that across the different edges, there are effects of pipeline and atlas on the strength of the functional connections, as included in Table S1, prompting further examination of these effects by considering both main and interaction effects.

**TABLE 4:** A summary of fixed effects, their levels, and notation for linear mixed effect models.

Fixed effect	Levels	Notation
Intercept (global mean)		μ
Pipeline	j=1,…,4	$\alpha_{j}$
Filtering	k=1,2	$\beta_{k}$
Atlas	ℓ=1,…,7	$\gamma_{\ell }$
Pipeline:Filtering Interaction	j⋅k=1,…,8	${\left(\alpha \beta \right)}_{\mathrm{jk}}$
Pipeline:Atlas interaction	j⋅ℓ=1,…,28	${\left(\alpha \gamma \right)}_{j\ell }$
Filtering:Atlas interaction	k⋅ℓ=1,…,14	${\left(\beta \gamma \right)}_{k\ell }$
Pipeline:Filtering:Atlas interaction	j⋅k⋅ℓ=1,…,56	${\left(\alpha \beta \gamma \right)}_{\mathrm{jk}\ell }$

The second model considers the same additive fixed effects, plus all possible interactions, and adjusts the random effects to include site‐specific random effects, as well as subject‐specific random effects nested within sites. We call this the full model, which is given by, for subject i and edge e,

$$\begin{array}{c} z_{\mathrm{ijk}\ell }^{\left(e\right)}=\left(\mu^{\left(e\right)}+\zeta_{m}^{\left(e\right)}+\eta_{i\left(m\right)}^{\left(e\right)}\right)+\alpha_{j}^{\left(e\right)}+\beta_{k}^{\left(e\right)}+\gamma_{\ell }^{\left(e\right)}+{\left(\alpha \beta \right)}_{\mathrm{jk}}^{\left(e\right)}\\ \;+{\left(\alpha \gamma \right)}_{j\ell }^{\left(e\right)}+{\left(\beta \gamma \right)}_{k\ell }^{\left(e\right)}+{\left(\alpha \beta \gamma \right)}_{\mathrm{jk}\ell }^{\left(e\right)}+\epsilon_{\mathrm{ijk}\ell }^{\left(e\right)} \end{array}$$

where $\zeta_{m}^{\left(e\right)}∼N\left(0,{\left(\sigma_{\zeta }^{\left(e\right)}\right)}^{2}\right)$ are site‐specific random effects that are independent across sites m and network edges e, $\eta_{i\left(m\right)}^{\left(e\right)}∼N\left(0,{\left(\sigma_{\eta }^{\left(e\right)}\right)}^{2}\right)$ are subject‐specific effects nested within sites, which are independent across sites m, subjects i, and network edges e, and $\epsilon_{\text{ijk}\ell }^{\left(e\right)}\overset{\text{iid}}{∼}N\left(0,{\left(\sigma^{\left(e\right)}\right)}^{2}\right)$ are the errors. Also, we consider a separate, third model that includes all of the effects of the full model, plus an additive scanner brand effect. The results are discussed in Supporting information S4.

### 2.4 Uniform Manifold Approximation and Projection of Heterogeneity Effects

Beyond using LMMs to quantify edgewise effects of pipeline, filtering, and atlas on the estimated FC network, we employ additional methods to further assess how these factors affect the replicability of a functional network. The first of these methods uses Uniform Manifold Approximation and Projection (UMAP) (McInnes et al. 2020). UMAP is a dimension reduction technique designed for visualization by searching for the closest low dimensional projection of the data that replicates the topological structure of the original data (McInnes et al. 2020). Our goal is to visualize the effects of interest on the functional networks in a succinct and comprehensible way, and graphically displaying that information in two dimensions is a natural solution.

We choose UMAP for dimension reduction as opposed to other methods such as principal component analysis (PCA) and *t*‐distributed stochastic neighbor embedding (*t*‐SNE) for a couple of reasons. With PCA, that would imply plotting only the first two principal components, which may not explain a large proportion of the overall variation in the data. *t*‐SNE, on the other hand, can also visually display the data when reduced to only two dimensions effectively, but it is regarded more for a focus on local patterns in the data (Marx 2024). UMAP strikes a better balance between retaining local information but demonstrating more global patterns across the entire dataset (Marx 2024), which aligns with our goals for the visualization of these effects.

To apply UMAP, we first subtract out the estimated random effects of scanning site and each subject ID nested within the scanning site from the full model for each edge to control for the repeated measurements per subject and the site effect; we then treat the Fisher's z‐transformed values of the 435 edges in the undirected network as a 435‐dimensional vector. Then, we apply UMAP and project the vector into two‐dimensional space using the umap package in R (Konopka 2023). Finally, we plot the coordinates for each point on the Cartesian plane. For each factor, the points are color‐coded by level, allowing us to visualize the differences by the spatial distribution of points.

### 2.5 Analysis of Intra‐Subject Network Variability

We examine how functional networks vary within subjects across different processing combinations by comparing resulting networks using two different metrics. The first metric that we use is the Frobenius norm of the difference between pairs of networks, which examines the differences in networks from an edgewise perspective by accumulating the differences between the functional connections. The second metric is network portrait divergence proposed by Bagrow and Bollt (2019), which is an information‐theoretic measure that captures the similarity of two networks based on the homogeneity of their topological characteristics. Luppi et al. (2024) have used portrait divergence for measuring intra‐subject variability across pipeline combinations for binarized FC networks.

For two FC networks $G,G^{\prime }\in {\mathbb{R}}^{30\times 30}$ where the (i,j)th element corresponds to the Fisher's z‐transformed correlation between ROI i and j, the Frobenius norm of the difference between G and $G^{\prime }$ is computed as,

$${\left\|G-G^{\prime }\right\|}_{F}=\sqrt{\sum_{i=1}^{30}\sum_{j=1}^{30}{\left|G_{\mathrm{ij}}-G_{\mathrm{ij}}^{\prime }\right|}^{2}}$$

This provides a measure of overall edgewise differences from G and $G^{\prime }$.

In comparison, portrait divergence compares the overall topology of the two graphs by computing the Jensen‐Shannon divergence between the distribution derived from their network portraits of G and $G^{\prime }$. As introduced by Bagrow and Bollt (2019), for an unweighted graph G, a network portrait of G is defined as an ℓ×k dimensional array B with the elements defined as,

(1) $$B{\left(G\right)}_{\ell ,k}\equiv \text{the number of nodes with}\;k\;\text{nodes}\;\mathrm{at}\;a\;\text{shortest path distance}\;\ell$$

for ℓ=0,1,…,d and k=0,1,…,N−1, where d is the graph's diameter and N is the number of nodes. For a weighted graph, the rows ℓ of $B\left(G\right)$ are formed by binning the shortest path lengths.

From the network portrait $B\left(G\right)$, a probability distribution $P_{G}$ is constructed as $P_{G}\left(k,\ell \right)={\mathrm{kB}}_{\ell ,k}/\sum_{c}n_{c}^{2}$ where $n_{c}$ represents the number of nodes in the connected component c. Each $P_{G}\left(k,\ell \right)$ represents the probability of choosing a pair of nodes at distance ℓ and for one of the two randomly chosen nodes to have k nodes at that distance. The network portrait divergence between two graphs G and $G^{\prime }$ is then defined as the Jensen‐Shannon divergence between $P_{G}$ and $P_{G^{\prime }}$, which is a symmetrized version of the Kullback–Leibler (KL) divergence, given by,

$$D_{\mathrm{JS}}\left(G,G^{\prime }\right)\equiv \frac{1}{2}\mathrm{KL}\left(P_{G}\;\|\;M\right)+\frac{1}{2}\mathrm{KL}\left(P_{G^{\prime }}\;\|\;M\right)$$

where $M=\frac{1}{2}\left(P_{G}+P_{G^{\prime }}\right)$ is the mixture distribution of $P_{G}$ and $P_{G^{\prime }}$. More detailed explanations can be found in Bagrow and Bollt (2019).

We note that unlike Luppi et al. (2024), we do not binarize the networks (e.g., restrict the networks to only denote edge presence/absence), but we compute the portrait divergence based on weighted graphs, using Fisher's z‐values as edge weights. Consequently, the networks remain fully connected, and the resulting portrait divergence measures how the deviations in the edge weights affect the network portraits for each subject, rather than the differences due to the presence or absence of edges.

For both the Frobenius norm and portrait divergence distribution approaches, we construct a baseline distribution using non‐parametric bootstrapping (Efron 1979) to provide a reference point. More specifically, our goal is to simulate from the distribution of the baseline network $P^{*}$, representing hypothetical data generated under a scenario where all participants are processed using an identical pipeline, filtering, and atlas combination. Formally, for a subject i with processing combination $c=\left(j,k,\ell \right)$, we define $z_{\mathrm{ic}}^{\left(e\right)*}$ as the adjusted Fisher's z‐transformed connectivity values $z_{\mathrm{ic}}^{\left(e\right)*}$ with all fixed effects removed:

$$z_{\mathrm{ic}}^{\left(e\right)*}=z_{\mathrm{ic}}^{\left(e\right)}-\mu \left(c\right)$$

where $\mu \left(c\right)=\mu \left(j,k,\ell \right)$ represents the fixed effects associated with the processing pipeline j, filtering k, and atlas ℓ combination. Thus $z_{\mathrm{ic}}^{*}={\left(z_{\mathrm{ic}}^{\left(e\right)*}\right)}_{e=1}^{435}∼P^{*}$ defines the (vectorized) connectivity networks under the scenario in which all subjects' data are processed identically.

We create B simulated datasets through bootstrapping, where each dataset $Z^{\left(b\right)*}$ consists of connectivity networks $z_{\mathrm{ic}}^{\left(b\right)*}∼P^{*}$ for $c\in \left\{\left(j,k,\ell \right);j=1,\ldots ,4,k=1,2,\ell =1,\ldots ,7\right\}$ and i=1,…,n individuals. To do so, we leverage the edgewise LMMs defined in Section 2.3. We first estimate $z_{\mathrm{ic}}^{*}$ by ${\hat{z}}_{\mathrm{ic}}^{*}$ where each element is given by

$${\hat{z}}_{\mathrm{ic}}^{\left(e\right)*}=z_{\mathrm{ic}}^{\left(e\right)}-\hat{\mu }\left(c\right)$$

with $\hat{\mu }\left(c\right)={\hat{\alpha }}_{j}^{\left(e\right)}\;+{\hat{\beta }}_{k}^{\left(e\right)}+{\hat{\gamma }}_{\ell }^{\left(e\right)}+{\hat{\left(\alpha \beta \right)}}_{\mathrm{jk}}^{\left(e\right)}+{\hat{\left(\alpha \gamma \right)}}_{j\ell }^{\left(e\right)}+{\hat{\left(\beta \gamma \right)}}_{k\ell }^{\left(e\right)}+{\hat{\left(\alpha \beta \gamma \right)}}_{\mathrm{jk}\ell }^{\left(e\right)}$ representing the estimated fixed effects from the full LMM.

Each bootstrapped dataset $Z^{\left(b\right)*}$ for b=1,…,B is constructed as follows:

1. Resample subjects: Draw a sample $I^{\left(b\right)}=\left\{i_{1}^{\left(b\right)},i_{2}^{\left(b\right)},\ldots ,i_{n}^{\left(b\right)}\right\}$ with replacement from the set of subjects i=1,…,n
2. Resample combinations for each subject: For each resampled subject $i^{\left(b\right)}$, draw a sample of processing combinations $C_{i^{\left(b\right)}}=\left\{c_{1}^{\left(b\right)},c_{2}^{\left(b\right)},\ldots ,c_{p}^{\left(b\right)}\right\}$ with replacement from the set of all combinations $\left\{\left(j,k,\ell \right);j=1,\ldots ,4,k=1,2,\ell =1,\ldots ,7\right\}$ where p=4×2×7.

The resulting dataset $Z^{\left(b\right)*}$ consists of p networks ${\hat{z}}_{\mathrm{ic}}^{*}$ for $c\in C_{i^{\left(b\right)}}$, for each resampled individual $i\in I^{\left(b\right)}$. We use B=100.

For both the original dataset Z and the bootstrapped datasets $Z^{\left(b\right)*}$, we compute pairwise differences between networks within each subject using both the Frobenius norm and the portrait divergence metrics. For each dataset, we estimate the empirical densities of within‐subject differences associated with specific processing factors. The baseline distribution is obtained by averaging the density curves from all B bootstrap replicates.

### 2.6 LMM and ComBat Harmonization for Controlling Site Effects

Since our dataset is collected from multiple sites, and site‐to‐site variations exist despite the use of the same protocol (ABIDE 2013), we control for site effects using two approaches: a linear mixed effects model approach with site‐specific random effects and ComBat harmonization proposed by Yu et al. (2018).

Several harmonization methods have been developed to address batch effects (Fortin et al. 2018; Johnson et al. 2007; Pomponio et al. 2020; Yu et al. 2018). Originally developed by Johnson et al. (2007) for gene expression data, ComBat harmonization has since been adapted for cortical thickness measurements (Fortin et al. 2018) and functional connectivity (Yu et al. 2018), and is one of the most widely used methods to correct for these batch effects. It combines empirical Bayes with a linear mixed effect model structure to control for batch effects while preserving the effects of interest. The location and scale effect of each site relative to each connectivity value are both estimated using empirical Bayes, along with the effects of interest. The ComBat‐adjusted connectivity values are an adjusted version of the model residuals, meaning the location effect and other effects are subtracted from the connectivity values while the scale effect is divided out, but the effects of interest are then added back in at the end of the procedure. This effectively preserves the effects of interest, while controlling for site variation in each connectivity value (Yu et al. 2018).

We report our findings in Sections 3.1, 3.4 in which site‐specific effects are adjusted using the first approach. We check the robustness of our conclusions by also conducting analysis using ComBat adjusted data. We use the lme4 package in R for the first approach, in which we fit a linear mixed effects model with site random effects. For ComBat harmonization of site effects, we use the neuroCombat package (Fortin 2024) in R. The results post‐ComBat harmonization are presented in Section 3.5 and Supporting information S3.

## 3 Results

**FIGURE 1:**
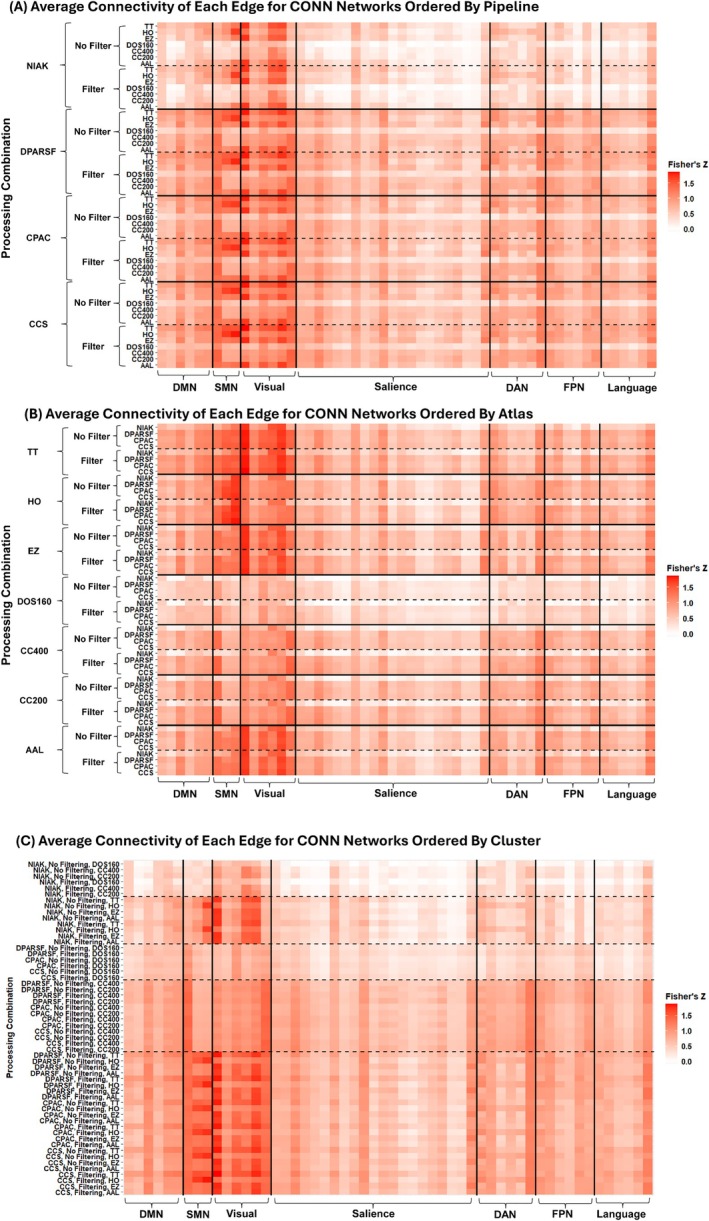
(A) Heatmap visualizing the mean Fisher's z‐transformed values for the edges within the networks across processing combinations, ordered by pipeline. (B) Heatmap visualizing the mean Fisher's z‐transformed values for the edges within the networks across combinations, ordered by atlas. (C) Heatmap visualizing the mean Fisher's z‐transformed values for the edges within the networks across combinations, ordered by cluster. The dashed lines distinguish the clusters.

As an initial exploratory step prior to any modeling, we plot the mean Fisher's z‐transformed correlation coefficients in a heatmap across all edges *within* each of the seven networks (54 edges) and processing combinations, shown in Figure 1. This provides a visualization of possible main and interaction effects and how they differ from one edge to another. To better visualize possible effects, we rearrange the rows of the heatmaps in three different configurations, giving the three panels seen in Figure 1. For ease of interpretation, in panel (A), the solid line breaks separate the preprocessing pipelines, and the dashed line breaks separate the band‐pass filtering scheme within a particular pipeline. The differences in brain parcellation (fixing pipeline and filtering scheme) are the rows in between any two line breaks. The primary ordering here is based on the pipeline, and most clearly demonstrates the possible pipeline effect. Of the four preprocessing pipelines, the NIAK pipeline deviates from the others the most. Specifically, the estimated strength of connections across all networks is weaker than those from other pipelines, but is least obvious in the visual network. The other three preprocessing pipelines are more consistent, although some variation still occurs.

In panel (B) of Figure 1, the solid line breaks now separate the parcellations, and the dashed line breaks still separate the band‐pass filtering scheme within a particular atlas. Now, the primary ordering is based on parcellation, and it is clearer to see a possible atlas main effect. The DOS160 atlas appears to consistently underestimate the strength of connections in comparison to the other atlases. The CC200 and CC400 atlases behave quite similarly, as do the AAL, EZ, HO, and TT atlases; however, the HO atlas shows some differences for the SMN and VN.

Finally, panel (C) orders the processing combinations based on the results from hierarchical clustering of the heatmap rows. We perform agglomerative hierarchical clustering with average linkage on the mean Fisher's z‐transformed values within each network, and after cutting the resulting dendrogram at a height of 1.5, have five clusters, which are denoted by the dashed lines in panel (C) of Figure 1. The cluster dendrogram can be found in Figure S1. The cluster results suggest three key points. First, this heatmap suggests there is little to no filtering effect, as no single cluster is defined based on filtering. Second, there is a clear distinction between the CC200, CC400, and DOS160 atlases versus the AAL, EZ, HO, and TT atlases, and this distinction is made much clearer in panel (C) of Figure 1 than in panel (B). Third, there appears to be a possible interaction effect between pipeline and parcellation. One example of this is that the top two clusters are defined solely by the NIAK pipeline's combination with the different atlases. For the other three pipelines, the clusters are defined by three groups of atlases: (1) AAL, EZ, HO, and TT, (2) CC200 and CC400, and finally (3) the DOS160 atlas. For the NIAK pipeline, the DOS160 atlas is instead grouped with the CC200 and CC400 atlases. The within‐network connectivity behavior is quite homogeneous within each cluster.

**FIGURE 2:**
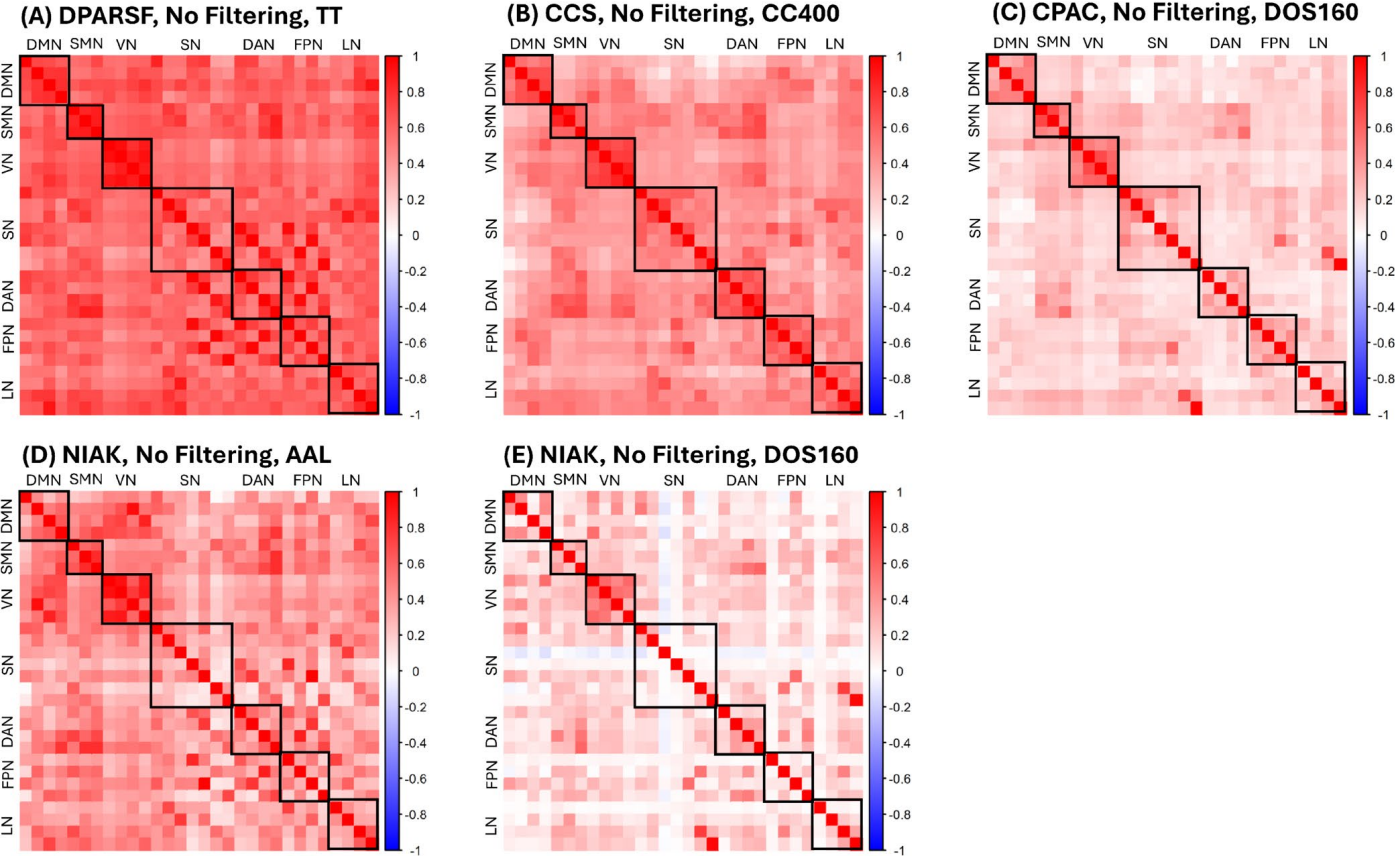
Average FC matrices across subjects are shown for five randomly selected processing combinations, one from each cluster identified in Figure 1 panel (C). As the effect of filtering does not define any of the five clusters, we fix the combinations to not perform band‐pass filtering before randomly sampling from each cluster. The seven functional networks are identified by the black boxes. The representative combinations exhibit considerable variation both within and between the various networks.

The heatmaps demonstrate significant *within‐network* variation between the different pipelines and parcellation schemes. We also examine possible variability in the context of the entire FC network. To explore this, we randomly sample one processing combination from each of the five clusters identified in Figure 1C. Since the choice of band‐pass filtering does not define any of the clusters, we fix the combinations to be only those that do not perform filtering. Figure 2 shows the average correlation matrices across subjects from the random sample. There is considerable variation both within and between the networks. We can reasonably expect that the correlations within‐network should be stronger than those between networks. However, some panels of Figure 2 show more of this block structure than others, with panels (B) and (C) being the most consistent in appearance.

The variability seen across edges and combinations in Figures 1 and 2 provides justification for further investigation of these effects. Section 3.1 discusses the linear mixed effect model results while Sections 3.2, 3.4 present additional results. Section 3.5 discusses the results when accounting for the batch effect of site using ComBat harmonization (Yu et al. 2018).

### 3.1 Edgewise Relative Effects of Processing Choices Using a Linear Mixed Effect Model

We now present the key results of the study, the estimated edgewise relative effects of preprocessing choices from the LMM fittings. The edgewise LMMs quantify the relative effects of pipeline, filtering, and atlas on the variability of FC estimates, as well as interaction effects. Our primary interest is examining how these effects manifest in the FC network; we are not interested in model selection or comparison. The additive effects model in Section 2.3 motivates exploration of possible interaction effects, and the full model with all higher‐order interactions is the model we examine and report on. In Table 5, we report the mean partial sum of squares values across the edges within each network as well as across all edges between any two networks from the Type III ANOVA edgewise full models. Type III refers to a partial sum of squares approach for unbalanced designs. In this approach, the sum of squares for each effect is computed via a series of reduced linear models (i.e., leaving one effect out at a time) (Mangiafico 2015). For example, the main effect of pipeline is assessed while controlling for filtering, atlas, and all interactions. The effects explaining the largest amounts of variation in the FC estimates are bolded.

**TABLE 5:** Mean partial sums of squares by network and between networks (BN) from the Type III ANOVAs are shown for the edgewise full models.

	DMN	SMN	VN	SN	DAN	FPN	LN	BN
**Pipeline**	331.63	750.85	354.16	378.51	300.06	700.75	370.80	750.85
Filter	2.92	8.22	1.46	4.29	1.40	3.59	1.86	1.05
**Atlas**	313.71	1680.93	1137.76	225.81	490.81	243.99	404.37	667.53
Pipeline:Filter	1.15	3.05	0.71	1.75	0.71	1.48	1.09	0.66
**Pipeline:** **A** **tlas**	46.35	63.65	18.41	42.76	57.73	21.22	24.48	26.80
Filter:Atlas	0.68	0.93	0.76	0.91	2.16	0.82	0.39	0.34
Pipeline:Filter:Atlas	0.28	0.36	0.28	0.36	0.79	0.32	0.19	0.15

**FIGURE 3:**
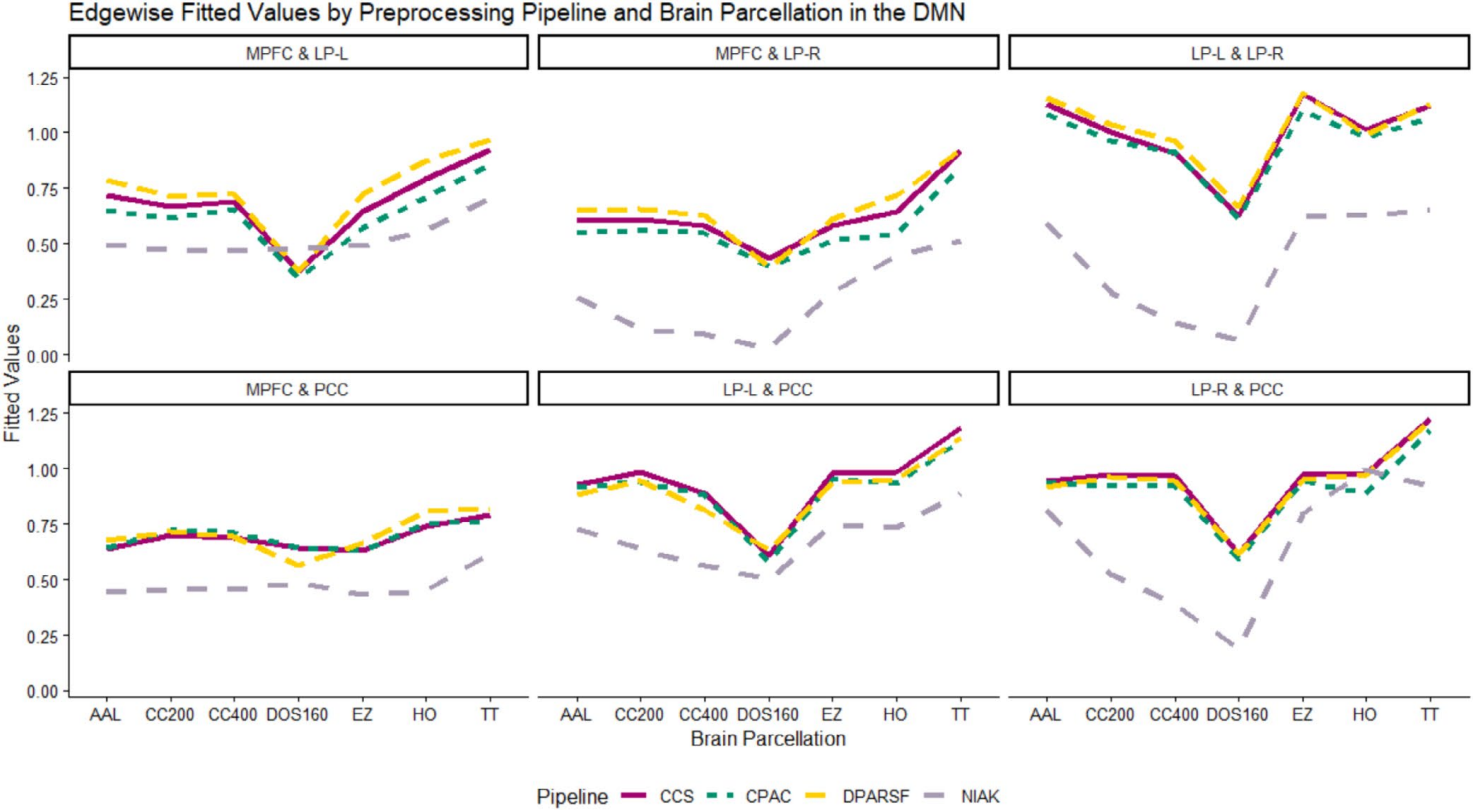
Interaction effects between preprocessing pipeline and brain parcellation are presented for the DMN edgewise full models. No interaction between these two factors implies that from one line segment to the next, the lines remain approximately parallel. The further the line segments deviate from parallel, the more influential the interaction is. The estimated Fisher's z‐transformed values after controlling for subject and site effects are shown on the y‐axis, the atlases are on the x‐axis, with a line for each pipeline. There is some interaction between the CCS, CPAC, and DPARSF pipelines with the different atlases, most notably in the MPFC and PCC edge, but the main source of the interaction is between the NIAK pipeline and the DOS160 and HO atlases.

The results in Table 5 show that, for the DMN, SN, and FPN, the average partial sum of squares value for pipeline is the largest, indicating that choice of pipeline explains the largest amount of variation in the strength of the functional connectivity for those networks. The atlas main effect explains substantial variation in the functional connectivity of each edge, with the partial sum of squares values being either the largest or second largest effect. As for filtering, relative to the other networks, the average partial sums of squares for the SMN edges are slightly larger, but in comparison to pipeline and atlas, filtering is not an impactful main effect, and neither are any of its interactions with the other factors. Additionally, the interaction between pipeline and atlas is the only interaction effect of note. According to Table 5, on average, the pipeline effect is not as strong *between networks* (BN) as it is *within* the networks, but the parcellation effect is still strong. We also examine the average effects between every pair of networks; we do not observe any systematic patterns across networks, showing the same overall trend with the atlas being the largest effect, followed by pipeline. We report individual between network edge results in Table S2.

To better visualize the effects of the pipeline–atlas interaction within a network, Figure 3 shows the fitted values from the full models for each edge after subtracting out the random effects plotted by atlas for the edges in the DMN, with a line for each pipeline. From one edge to another, there is little interaction between the CCS, CPAC, and DPARSF pipelines and atlas, with the main exception being in the edge between the MPFC and PCC where the DPARSF pipeline estimates stronger connections in some atlases but weaker connections in others. The main source of the interaction is between the NIAK pipeline and the various atlases. When the NIAK pipeline is combined with either the HO or DOS160 atlases especially, some functional connections are estimated to be higher than the NIAK pipeline otherwise would when combined with any of the other atlases.

From the results in Figures 1, 2, 3 and Table 5, the NIAK pipeline is a driving force behind the variability in FC estimates, both as a main effect and as an interaction effect with atlas. To better quantify how influential the NIAK pipeline is, we repeat the edgewise linear mixed effects modeling but exclude the samples using the NIAK pipeline. The mean partial sums of squares within and between networks from the full models, excluding the samples using the NIAK pipeline, are shown in Table 6. We find that the pipeline main effect is drastically reduced overall compared to the models with the NIAK pipeline shown in Table 5. However, in Table 6, the pipeline is more influential for the LN, FPN, and BN compared to the other networks, which differs from the pattern we observe in Table 5 where the SMN network has the largest partial sum of squares value for the pipeline across all networks. The main effect of band‐pass filtering is more pronounced for all edges after excluding the NIAK samples.

**TABLE 6:** Mean partial sums of squares by network and between networks (BN) from the Type III ANOVAs are shown for the edgewise full models, excluding the NIAK pipeline.

	DMN	SMN	VN	SN	DAN	FPN	LN	BN
Pipeline	9.01	11.56	7.40	6.36	9.22	16.94	21.64	16.00
Filter	3.88	10.93	1.95	5.71	1.86	4.77	2.47	1.39
Atlas	234.82	1224.46	878.65	194.02	460.17	213.53	370.61	552.67
Pipeline:Filter	0.17	0.30	0.22	0.31	0.24	0.28	0.47	0.31
Pipeline:Atlas	3.63	6.38	1.22	2.97	2.99	0.86	2.20	2.85
Filter:Atlas	0.90	1.24	1.01	1.21	2.87	1.09	0.52	0.45
Pipeline:Filter:Atlas	0.05	0.05	0.03	0.05	0.07	0.05	0.06	0.04

As filtering is not an impactful effect, we proceed with the remaining analysis for the pipeline and parcellation effects only, and discuss our findings in Sections 3.2, 3.4.

### 3.2 Functional Network Block Structure by Pipeline and Parcellation

We investigate which pipeline‐atlas combinations yield stronger block structure in the estimated connectivity networks, particularly in alignment with the reference block structure where each block corresponds to one of the seven functional networks. We do not consider the filtering effects, as filtering contributes minimally to variation in the estimated networks. To quantify the strength of alignment, we compute the difference between the average within‐network correlations and average between‐network correlations.

The results are presented in Table 7, with associated standard errors in parentheses. Larger values indicate combinations producing a stronger block structure; the five largest values in Table 7 are bolded. The processing combination resulting in the strongest alignment with the reference block structure is the CPAC pipeline with the CC400 atlas. Overall, with the exception of the NIAK pipeline, the CC200, CC400, and DOS160 atlases are consistently associated with stronger alignment across the network, where the within‐network connections are on average 0.2 larger (on a correlation scale) than between‐network connections. Results for the filtered combinations are qualitatively similar, though pipelines that perform filtering (CCS, CPAC, and DPARSF) generally exhibit slightly larger values, indicating modestly stronger preservation of the network block structure.

**TABLE 7:** Differences between average within‐network and between‐network correlation by pipeline and atlas (unfiltered samples), with the five largest values bolded.

	CCS	CPAC	DPARSF	NIAK
AAL	0.0652 (0.0015)	0.0833 (0.0016)	0.0632 (0.0015)	0.0080 (0.0022)
CC200	0.1832 (0.0013)	**0.2166** (0.0013)	**0.1820** (0.0013)	0.0271 (0.0021)
CC400	**0.1997** (0.0013)	**0.2346** (0.0014)	**0.1966** (0.0014)	0.0269 (0.0021)
DOS160	0.1593 (0.0018)	0.1683 (0.0019)	0.1563 (0.0018)	0.0356 (0.0021)
EZ	0.0746 (0.0015)	0.0916 (0.0016)	0.0734 (0.0015)	0.0211 (0.0021)
HO	0.0640 (0.0016)	0.0790 (0.0017)	0.0643 (0.0016)	0.0178 (0.0022)
TT	0.0620 (0.0015)	0.0800 (0.0016)	0.0558 (0.0014)	0.0062 (0.0022)

*Note:* Differences between the average within‐network and between‐network correlations are presented for all pipeline and atlas combinations. First, the site‐specific and subject‐specific random effects are subtracted out from the Fisher's z‐transformed values before inverting the z‐values back to correlation r values. Then, for each pipeline and atlas combination (we choose the unfiltered samples, but do not consider filtering here as it is an unimportant effect), we find the mean FC matrix across subjects. Finally, we compute the average within‐network correlations, and between‐network correlations (using the upper triangle of the network), and take the difference. The standard errors are reported in parentheses next to each value. Larger values indicate a stronger block structure to the average FC matrix for that pipeline–atlas combination.

### 3.3 Preprocessing Pipeline Effect

#### 3.3.1 UMAP Visualization of Pipeline Effect

**FIGURE 4:**
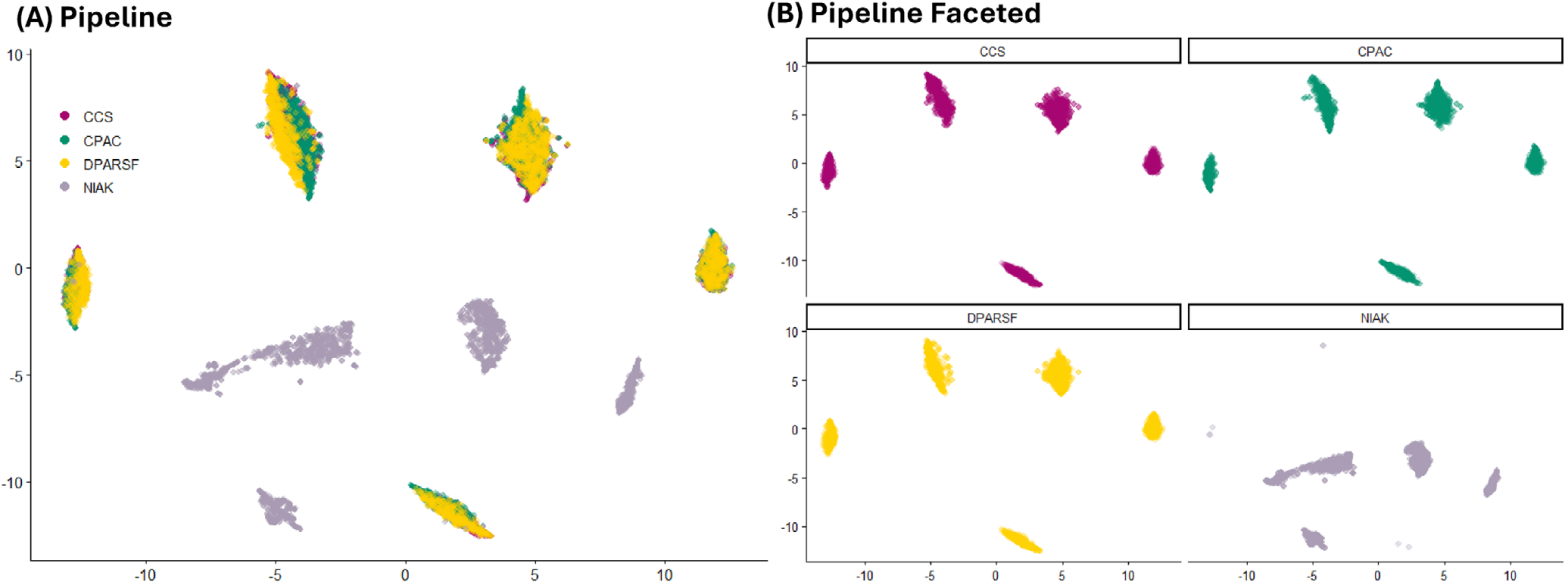
The UMAP visualization results for the full network (435 edges) broken down by pipeline. (A) The UMAP is shown where each color indicates a different pipeline. (B) The UMAP results when faceted by pipeline. The results demonstrate how distinctly different the NIAK pipeline is. The CCS, CPAC, and DPARSF pipelines all look relatively similar, whereas there is no overlap between the NIAK pipeline and the others.

The UMAP visualizations and the examination of Frobenius norms and portrait divergence further highlight the difference of the NIAK pipeline from the other three. UMAP visualizations for the entire network (435 edges) with the points colored by pipeline are shown in Figure 4. We also use UMAP on just the within‐network, as well as the between‐network edges, and the visualizations colored by pipeline and atlas are in Supporting information S3. In panel (A) of Figure 4, the points corresponding to the CCS, CPAC, and DPARSF pipelines appear to overlap, but the NIAK pipeline appears separately. In panel (B) when the projection is faceted according to pipeline, this distinction becomes even clearer, as the NIAK pipeline exists in a distinct portion of the Cartesian plane compared to the other three. Panel (B) of Figure 4 also confirms the suspected overlap seen in panel (A) for the other three pipelines. The one cluster where there is a slight bit of distinction is in the top‐most cluster second from the left, in which the CPAC and DPARSF pipelines do appear to differ slightly.

#### 3.3.2 Intra‐Subject Network Variability due to Pipeline Differences

**FIGURE 5:**
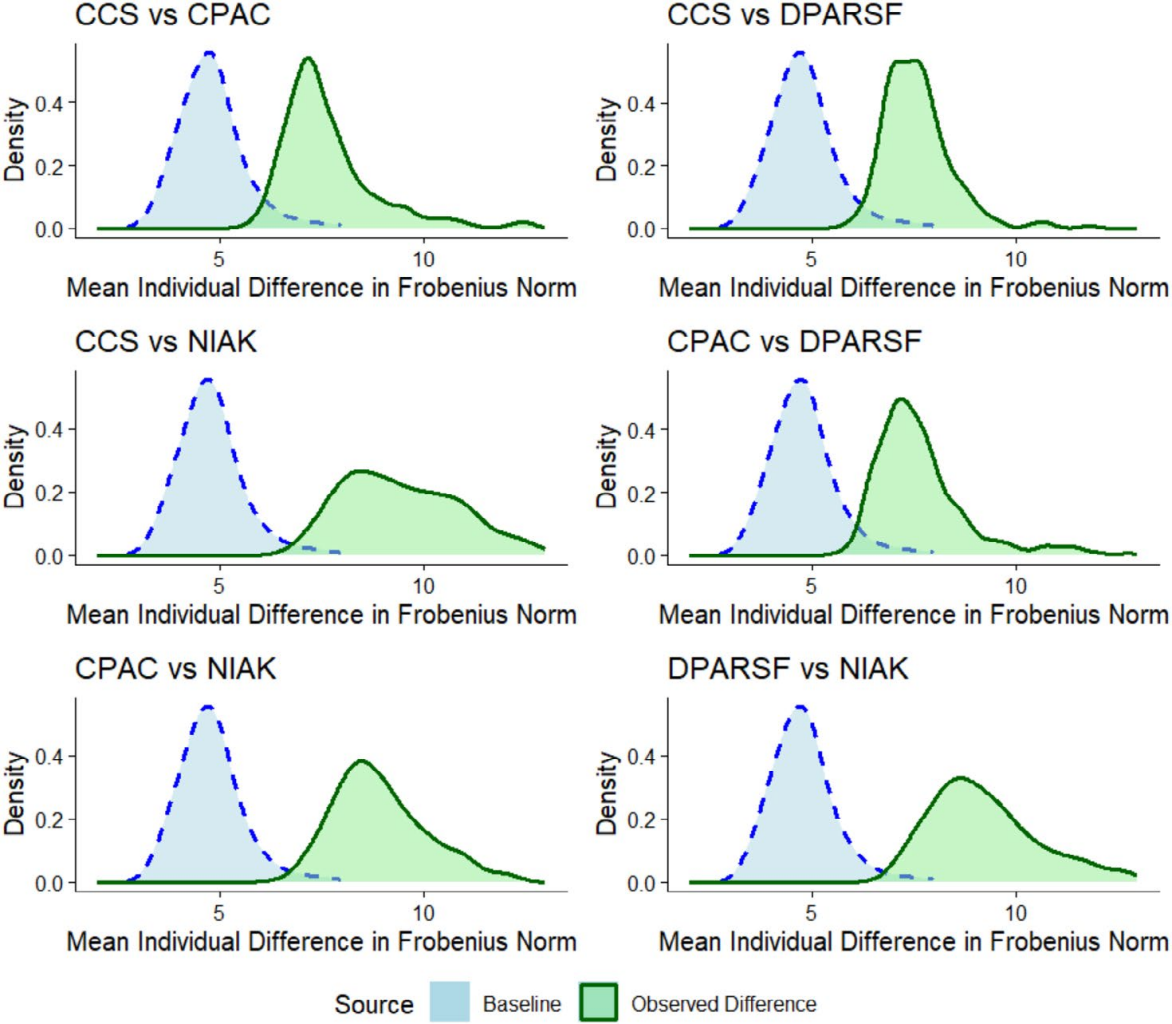
Density plots comparing the mean Frobenius norm values across individuals for different pairwise pipeline comparisons (e.g., CCS vs. CPAC) with the bootstrapped baseline density. The baseline curve represents the density of the average difference in Frobenius norms that we can expect with subject and site level variation, but zero pipeline variation. The further away the real data density is from the baseline curve indicates the larger amount of variation that can be attributed to the overall network if a subject is processed with one pipeline versus the other. The comparisons involving the NIAK pipeline are the furthest away from the baseline, while the other three pipelines still exhibit differences, but their modes are much closer to the baseline and the distributions have smaller variances.

Figure 5 shows the density of the difference in Frobenius norms for each pipeline pairwise comparison between the bootstrapped baseline curve (blue) and the observed data (green). For more information on the final baseline curves shown after bootstrapping, see Figures S5 and S6. The plots can be interpreted in the following way: the blue curve represents the distribution of the Frobenius norm of the differences in the networks we can expect with only subject and subject nested within scanning site variability, or the distribution we can expect from multi‐site data all processed under the identical pipelines. Therefore, the green curve, or the observed data, represents the shift in variation that is induced when processing the data for the same set of subjects using two different pipelines. Taking the CCS versus CPAC comparison as an example, the observed data shifts the mode by an average difference in the Frobenius norm from a 4.8 baseline mode to about 7, but the shapes of the distributions are very similar. This suggests that while there are edgewise differences that accumulate across the networks between the CCS and CPAC pipelines, it is a fairly consistent shift. In contrast, the comparisons involving the NIAK pipeline all have modes that are farthest away from the baseline and have larger variances, implying that the same subjects processed with NIAK versus any other pipeline will have more substantial network‐wide variation, leading to less consistent results.

**FIGURE 6:**
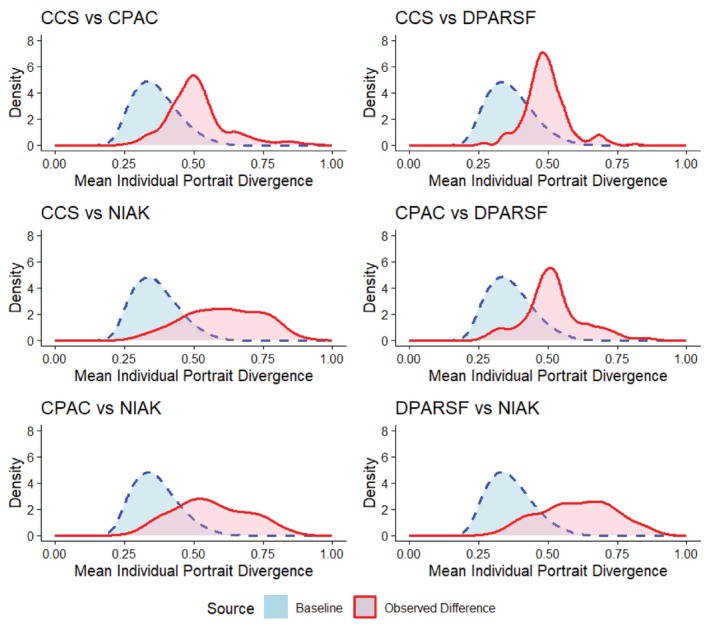
Density plots comparing the mean portrait divergence values across individuals for different pairwise pipeline comparisons (e.g., CCS vs. CPAC) with the bootstrapped baseline density. The baseline curve represents the density of the average portrait divergence we can expect with subject and site level variation, but zero pipeline variation. The further away the real data density is from the baseline curve indicates the larger amount of variation that can be attributed to the overall network if a subject is processed with one pipeline versus the other. The comparisons involving the NIAK pipeline have a much flatter density suggesting a higher level of variation in the network portraits, while the other three pipelines still exhibit differences, but their modes are much closer to the baseline and the distributions have smaller variances.

As for the portrait divergence comparisons, Figure 6 shows the baseline curves in blue, and the observed portrait divergence curves in red. As the support of the portrait divergence measure is restricted between zero and one, the densities are more compact than they are for the comparisons using Frobenius norms. Other than the support of the measures being different though, the general interpretation of Figure 6 remains the same as described for Figure 5. For the comparisons involving NIAK, the distributions of portrait divergence values are much flatter than that of the baseline, and all have non‐zero densities at values very close to one, meaning the same set of samples processed with NIAK versus every other pipeline have instances where the network portraits are nearly maximally different. The comparisons between the other three pipelines still represent a shift in the network portraits, but there is more overlap with this network measure than that of the Frobenius norms. When considering the entire network topology in calculating distance/divergence, the other three pipelines produce more similar networks to one another than the Frobenius norm results otherwise suggest.

### 3.4 Brain Parcellation Effect

#### 3.4.1 UMAP Visualization of Parcellation Effect

**FIGURE 7:**
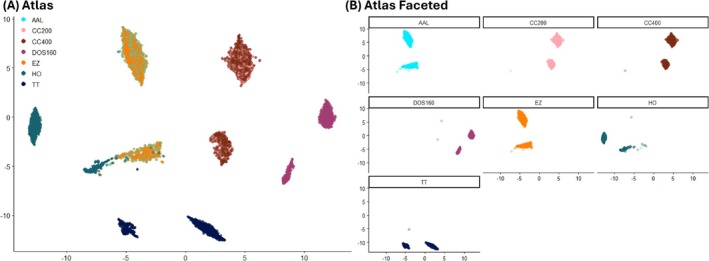
The UMAP visualization results for the full network (435 edges) broken down by atlas. (A) The UMAP is shown where each color indicates a different atlas. (B) The UMAP results when faceted by atlas. The results demonstrate the consistency between the AAL and EZ atlases, as well as between the CC200 and CC400 atlases. The DOS160, HO, and TT atlases each occupy distinct portions of the Cartesian plane. The clusters near the center of the space plus the bottom left correspond to the NIAK pipeline and further demonstrate the interaction effect between the NIAK pipeline and the various parcellations.

For parcellation effects, the UMAP results are shown in Figure 7 with the points overlaid on top of each other in panel (A), and the results when faceted by atlas in panel (B). The CC200 and CC400 atlases generally agree with one another, which is reasonable to expect as both atlases are created using the same procedure, just with different numbers of parcels. Additionally, the AAL and EZ atlases are quite similar in their two‐dimensional FC network projections, which is also not surprising considering how similar the atlases are both in terms of the number of parcels and the boundaries defining them. The DOS160, HO, and TT atlases all occupy distinct portions of the space. In comparison with Figure 4, each atlas belongs to two clusters, and the second cluster always corresponds to the NIAK pipeline, further demonstrating how drastically different the results are when using the NIAK pipeline, regardless of atlas choice.

#### 3.4.2 Intra‐Subject Network Variability due to Atlas Differences

**FIGURE 8:**
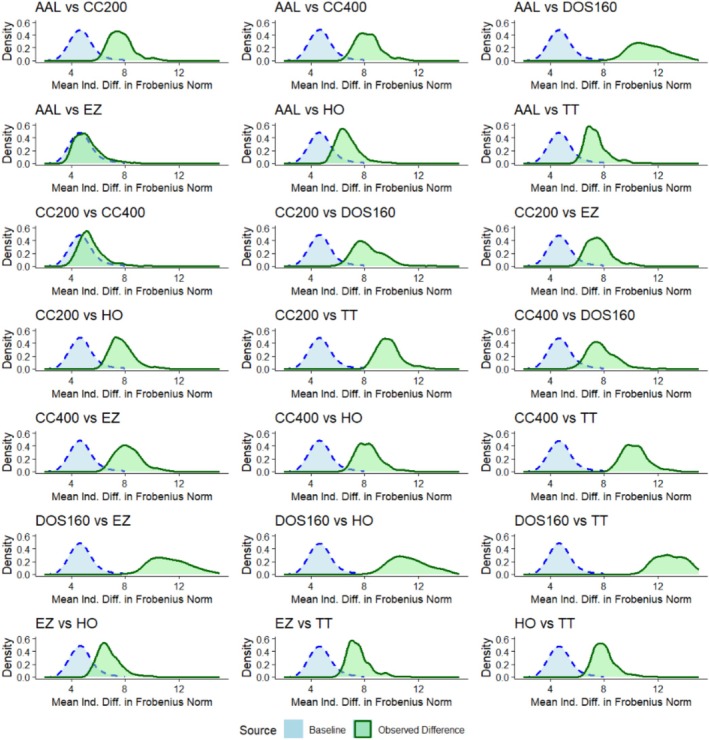
Density plots comparing the mean Frobenius norm values across individuals for different pairwise atlas comparisons (e.g., AAL vs. DOS160) with the bootstrapped baseline density. The baseline curve represents the density of the average difference in Frobenius norms that we can expect with subject and site level variation, but zero atlas variation. The further away the real data density is from the baseline curve indicates the larger amount of variation that can be attributed to the overall network if a subject is processed with one atlas versus the other. The comparisons involving the DOS160 are the furthest away from the baseline. The AAL and EZ comparison, and the CC200 and CC400 comparison show a high degree of overlap with the baseline, suggesting a high level of consistency between subjects processed with either one of these combinations.

The Frobenius norm results in Figure 8 further demonstrate the agreement between the AAL and EZ, as well as the CC200 and CC400 atlases. There is near perfect overlap between the AAL and EZ atlas comparison with the constructed baseline curve, and a strong degree of overlap between the CC200 versus CC400 comparison and the baseline. The atlas most clearly different from the rest according to this measure is the DOS160 atlas, as all comparisons involving it have little to no overlap with the baseline. The comparisons involving DOS160 that are closest to the baseline are with CC200 and CC400. According to this measure, the HO and TT atlases are more dissimilar from the AAL and EZ atlases than the clustering results from Figure 1C suggest, but more similar than the UMAP results in Figure 7 show. This demonstrates the importance of examining variability in these networks in a variety of ways, in order to better understand the magnitude of differences that may exist. There is no single way to exactly quantify how different these factors are from one another.

**FIGURE 9:**
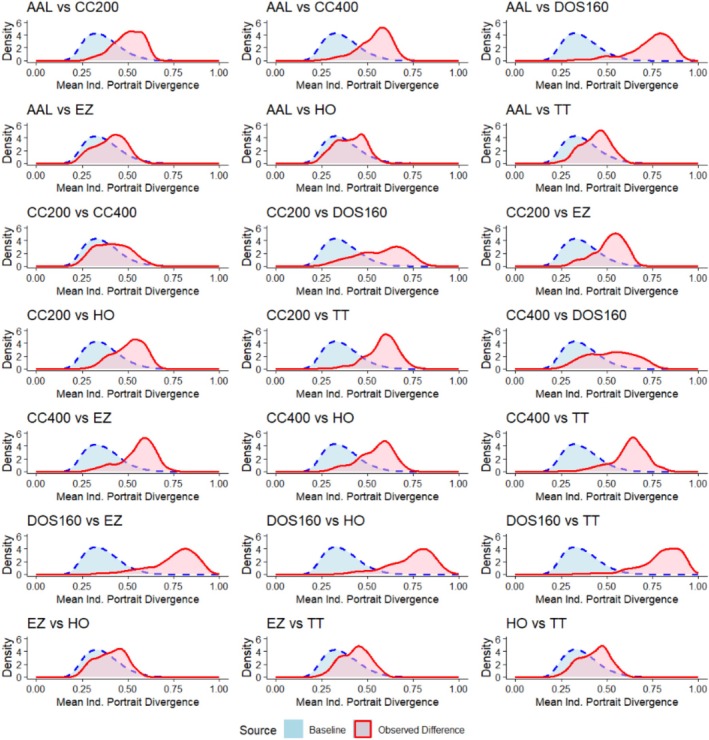
Density plots comparing the mean portrait divergence values across individuals for different pairwise atlas comparisons (e.g., AAL vs. DOS160) with the bootstrapped baseline density. The baseline curve represents the density of the average portrait divergence we can expect with subject and site level variation, but zero atlas variation. The further away the real data density is from the baseline curve indicates the larger amount of variation that can be attributed to the overall network if a subject is processed with one atlas versus the other. The comparisons involving the DOS160 are left skewed, and are the furthest away from the baseline. Based on portrait divergence, there is a high level of consistency in the network portraits between the AAL, EZ, HO, and TT atlases, as well as between the CC200 and CC400 atlases.

Lastly, the portrait divergence results in Figure 9, show some similarities to the Frobenius norm results and some differences. For the similarities, the AAL and EZ, and CC200 and CC400 comparisons are once again overlapping with the baseline curve, indicating consistency in the network portraits for the same set of subjects with FC matrices parcellated using either of these pairs of atlases. Also, the DOS160 atlas is most distinct from the other atlases on average. As the DOS160 parcellation does not partition the entire brain in the same way the other atlases do, and instead provides localized spheres for each region, this result is not surprising. These findings should not detract from the use of a parcellation such as DOS160 for FC analysis if it strongly aligns with a study's research goals, but rather inform the community of some downstream implications that are to be expected when parcellating the brain according to different mechanisms. The key difference of note with the portrait divergence results in Figure 9 compared to the Frobenius norm findings is the congruence between not just the AAL and EZ atlases, but across AAL, EZ, HO, and TT atlases. When considering the divergence in network portraits, all four atlases behave quite similarly to the expected baseline variation in network portraits for these data.

### 3.5 Reevaluation of Effects Post‐ComBat Harmonization

We also assess the impact of pipeline, filtering, and parcellation on FC replicability after performing ComBat harmonization for the batch effect of site. In doing this, we account for having multi‐site data in two ways, which better allows for an accurate assessment of the replicability effects while controlling for site effect. For the heatmap examining the mean Fisher's z‐transformed values across all combinations, the results after ComBat harmonization for the site effect are unchanged. The same generally goes for the linear mixed effects models, with the results shown in Table 8. The random intercept for subject ID nested within site is no longer necessary given the batch effect adjustment, so the random intercept for subject ID is only to account for the repeated measurements for each subject across the combinations. For the main and interaction effects, the results remain consistent after ComBat harmonization, with pipeline, atlas, and the two‐way interaction between pipeline and atlas all being notable effects. As for UMAP, the two‐dimensional projection after batch effect correction shows up slightly differently within the space, but the same patterns hold as those described in Sections 3.3 and 3.4. The post‐ComBat UMAP results for pipeline and atlas are shown collectively in Figure S4.

**TABLE 8:** Mean partial sums of squares within‐ and between‐networks (BN) from the Type III ANOVAs for the edgewise full models post‐ComBat harmonization (Yu et al. 2018).

	DMN	SMN	VN	SN	DAN	FPN	LN	BN
Pipeline	326.03	741.41	350.72	377.98	298.51	696.86	367.75	137.38
Filter	2.98	8.18	1.52	4.39	1.48	3.63	1.89	1.08
Atlas	315.10	1680.38	1136.21	225.69	487.77	243.73	405.89	667.81
Pipeline:Filter	1.21	3.16	0.75	1.84	0.77	1.55	1.13	0.70
Pipeline:Atlas	47.48	65.48	19.04	43.68	59.10	21.97	25.36	27.60
Filter:Atlas	0.72	0.99	0.79	0.97	2.25	0.86	0.41	0.36
Pipeline:Filter:Atlas	0.29	0.38	0.30	0.38	0.83	0.34	0.20	0.16

## 4 Discussion

We use seven resting‐state networks as tools for studying the effects of different preprocessing combinations on the replicability of functional connectivity estimates. Our findings demonstrate that different choices of preprocessing pipeline and brain parcellation result in network variability, while applying a 0.01–0.1 Hz band‐pass filter has little effect. Also, we observe a strong interaction effect between pipeline and the choices of parcellations, where certain pipeline and atlas combinations consistently result in higher or lower average connectivity. These effects persist even after controlling for the additional variation from neuroimaging sites and within individual subjects by regressing out these random effects in the linear mixed effects model or using ComBat harmonization.

More specifically, our findings demonstrate that the use of the NIAK pipeline results in noticeably weaker connections amongst the ROIs within and between the seven functional networks, and when compared with other pipelines, gives inconsistent functional network estimates, both within and between subjects. While NIAK is no longer maintained, the pipeline has been used for the preprocessing of rs‐fMRI data (Orban et al. 2015; Therriault et al. 2019). While other studies find significant variation across many pipelines (Luppi et al. 2024; Shirer et al. 2015), the observed variation in the current study is mostly attributed to the comparison of the NIAK pipeline with the other three pipelines. When the NIAK pipeline is removed from the analysis, there is a drastically reduced pipeline effect. This suggests that the CCS, CPAC, and DPARSF pipelines do still lead to variation in FC estimates, but are more consistent with one another. These three pipelines should lead to greater replicability of results if two studies use any two of the CCS, CPAC, and DPARSF pipelines to preprocess the data versus one study using the NIAK pipeline instead.

Fortunately, there is movement towards standardizing preprocessing procedures and data management practices within neuroimaging, which is crucial for the reproducibility and replicability of results. While no one pipeline is superior to another, the transformations that occur during preprocessing affect downstream results regardless of the methodologies chosen, and further agreement on the tools to be used makes for better comparisons across studies. Software applications that streamline preprocessing choices such as fMRIPrep (Esteban et al. 2019), data organization and structure following the Brain Imaging Data Structure (BIDS) (Gorgolewski et al. 2017) as well as OpenNeuro (Markiewicz et al. 2021) and other resources for the sharing of neuroscience data are becoming mainstream. Their continued use further promotes confidence in scientific results, as potential replicability effects from preprocessing pipeline can be reduced.

While the preprocessing pipelines we examine in the current study (CCS, CPAC, DPARSF, and NIAK) reflect commonly used workflows at the time of the ABIDE release, contemporary studies increasingly rely on standardized pipelines such as fMRIPrep (Esteban et al. 2019). Early preprocessing steps differ modestly across ABIDE (2013) pipelines, including removal of initial volumes and the application of slice timing correction, whereas fMRIPrep performs slice timing correction but does not remove initial volumes by default. For motion correction, fMRIPrep uses MCFLIRT in FSL in contrast to AFNI‐ and SPM‐based implementations in ABIDE. Greater differences arise in intensity normalization, where CCS and CPAC apply global mean scaling, NIAK uses median‐based scaling, and DPARSF applies none, while fMRIPrep performs N4 bias field correction on anatomical images without global rescaling of functional data. ABIDE pipelines vary between boundary‐based and rigid‐body co‐registration approaches, whereas fMRIPrep applies boundary‐based registration. Spatial normalization also differs substantially, with ABIDE pipelines using FLIRT/FNIRT, ANTs, DARTEL, or CIVET, while fMRIPrep uses ANTs and composes all spatial transforms into a single resampling step. The largest differences occur in nuisance modeling and filtering: ABIDE pipelines incorporate nuisance regression and band‐pass filtering within preprocessing, whereas fMRIPrep performs neither step and instead outputs standardized confound regressors, including motion parameters, tissue signals, and framewise displacement, among others, for downstream analysis. Finally, spatial smoothing is applied in some ABIDE pipelines (DPARSF and NIAK) but not by default in fMRIPrep (Esteban et al. 2019).

The software tools underlying fMRIPrep do overlap with those used in several ABIDE pipelines, particularly CPAC, which also incorporates ANTs‐based normalization and boundary‐based registration. Thus, while the ABIDE pipelines are less commonly used in current practice, they are not entirely distinct from contemporary approaches. However, the results presented here demonstrate that differences in both preprocessing and denoising strategies can meaningfully impact downstream analyses. Given the widespread adoption of fMRIPrep as a standardized preprocessing framework, future work should directly compare fMRIPrep outputs with those obtained from pipelines such as CCS, CPAC, and DPARSF to further evaluate the extent to which these methodological differences influence replicability.

The primary motivation behind band‐pass filtering is to eliminate noisy wavelengths and enhance the strength of the underlying signal. We do not observe a significant impact of 0.01–0.1 Hz band‐pass filtering on FC network estimation within or between the seven networks, in contrast to the findings from Shirer et al. (2015). However, Shirer et al. (2015) apply band‐pass filtering prior to nuisance signal regression and exclusively use FSL for their preprocessing pipeline, whereas the ABIDE developers apply it afterwards and only some pipelines use select FSL functions, which may be contributing to these differences. Since we use Pearson correlation as the measure of connectivity, our findings agree with Sala‐Llonch et al. (2019) though, as they also find that regardless of parcellation scheme, there are similar results between a 0.005 and 0.096 Hz filter compared to no filter for Pearson correlation. Also, we recognize that some of the lack of effects may be a result of a lack of filtering within the NIAK pipeline (elaborated on in Section 2.1.2). We do note that when repeating the linear mixed effect model analysis after excluding the NIAK pipeline samples, the main effect of filtering is more pronounced across all edges, which does provide evidence for an effect of filtering when restricting our samples to the three pipelines that are more consistent with one another.

The limited impact of band‐pass filtering we observe is also consistent with known limitations of frequency‐based filtering for typical fMRI acquisition rates. For standard repetition times (e.g., TR = 2 s), physiological signals such as cardiac and respiratory cycles are subject to aliasing, limiting the extent to which these components can be effectively removed through band‐pass filtering alone (Cordes et al. 2001). Additionally, band‐pass filtering reduces the effective degrees of freedom in the time series, which may introduce increased variability in connectivity estimates, particularly for shorter acquisitions (Birn 2023). In the ABIDE dataset, scan durations vary across sites (mean = 6.3 min, SD = 2.3 min), and most sites use a 2 s TR. Nevertheless, we observe minor differences between filtered and unfiltered connectivity estimates, suggesting that the reduction in degrees of freedom does not materially impact the stability of functional connectivity in the current study.

Additionally, there is no formal consensus about the exact parcellation for the human brain (Arslan et al. 2018; Bijsterbosch et al. 2020; Eickhoff et al. 2018; Luppi et al. 2024; Messé 2020; Papo et al. 2014; Sala‐Llonch et al. 2019). Large‐scale functional network definitions are not uniquely specified, as the number and composition of networks can vary depending on the methodological choices made to create the network boundaries. In our comparison of parcellations, even when enforcing objectivity in determining which regions in each atlas form the ROIs of the seven networks used, there are challenges, and these challenges manifest in the resulting FC estimates in the network. From this perspective, it can be problematic to make comparisons across results in the literature which study the same functional network but apply different parcellation strategies. Thus, while the seven‐network framework we use from CONN provides a useful and widely adopted reference, our results should be interpreted as conditional on this particular representation of functional organization.

As the body of neuroimaging literature continues to grow, a natural goal is to synthesize results across studies and reach a consensus about how brain networks function and interact with one another. This task becomes increasingly difficult when researchers use different parcellation strategies, and especially when they are not explicit about it. Even more fundamental of an issue is when researchers are trying to replicate or reproduce another study's results. If researchers are not explicit regarding the choices made when defining ROIs and forming a functional network, it is very difficult for a different research team to reproduce results with the same data or to replicate results using new data and methods.

There are many atlases in circulation, and the optimal choice depends on the research question; some studies require fine‐grained parcellations, while others could benefit from using coarser parcellations. Given that this choice of parcellation does have downstream effects on studying functional networks, we strongly encourage that researchers determine their parcellation choice before examining any data and clearly document their reasoning. In addition, any specific choices in how that parcellation is used, such as what parcels constitute ROIs for a functional network, also need to be explicitly stated. In this study, we use an entirely separate atlas validated on a different sample of almost 500 subjects, and strict decision rules are implemented and explained for ensuring objectivity in region selection. This is one of many possible ways for introducing more open science‐based practices in functional neuroimaging research.

Beyond the need for future work on incorporating direct comparisons between standardized tools such as fMRIPrep and the ABIDE pipelines on downstream analysis, we address some additional limitations and future research directions. First, although the sample consists of neurotypical participants with a mean age in adolescence, the brain parcellations we employ are derived from adult populations. Given that functional brain organization continues to develop throughout adolescence (Fair et al. 2009), this mismatch may introduce age‐related biases in the definition of network boundaries and, consequently, in downstream connectivity estimates. We note that, while we use the CONN atlas as a consistent template to compare the ABIDE parcellations, the parcellations distributed with the ABIDE data are also primarily derived from adult populations, reflecting a broader limitation of the available resource. The use of developmentally appropriate parcellations or data‐driven approaches to better capture age‐specific functional organization and evaluate how such choices interact with processing decisions is a valuable direction for future work.

Second, our analysis is restricted to neurotypical controls, which provides a relatively stable setting for isolating the effects of processing choices but does not capture the additional heterogeneity present in clinical populations. ASD is characterized by substantial variability in both behavior and brain function (Di Martino et al. 2014), and the ABIDE (2013) dataset provides rs‐fMRI scans for a near equal number of individuals with ASD as it does for neurotypical individuals. Future research should therefore extend these analyses to clinical cohorts, including ASD, to evaluate how processing decisions interact with population heterogeneity and to assess their impact on the replicability of connectivity findings. Such work may also improve the reliability of connectivity‐based biomarkers in clinical populations.

Third, for the analysis of parcellation‐choice effects, we have created an intermediate mapping from atlas parcels to fixed network‐level ROIs based on the CONN atlas, because the ABIDE parcellations are not organized according to the functional networks considered in this study. This introduces an approximation that depends on the spatial resolution and boundaries of the underlying parcellation. In particular, coarser atlases, such as HO and TT, may provide a less precise representation of the network ROIs under this mapping, whereas finer parcellations, such as CC400, may better preserve network structure. This can influence some parcellation‐related results in the paper. For example, the differences in within‐network and between‐network connectivity estimates across parcellations in Table 7 may partly reflect how closely each atlas approximates the CONN network ROIs under the overlap‐weighting scheme, rather than the intrinsic superiority of one parcellation over another. As a result, the differences we find across parcellations show how variation in parcel definitions propagates to connectivity estimates through the overlap weights and does not establish superiority of one atlas over another. While higher‐resolution atlases may better approximate the CONN network structure, this comes with potential trade‐offs, including a greater multiple testing burden.

While our approach captures some aspects of how parcellations affect functional network variability, a complementary perspective is to consider variability in the definition of large‐scale functional networks themselves. For example, there are other network‐based atlases (e.g., Power et al. 2011; Shirer et al. 2012; Yeo et al. 2011) that provide alternative definitions of several large‐scale functional networks, including definitions for some of the networks in the CONN networks atlas. These atlases differ from those in the ABIDE dataset, which are broader parcellations not inherently organized into functional networks. The network‐based atlases can operate directly on voxel‐level data and compare network definitions without an intermediate parcellation mapping. Future work comparing the replicability of FC estimates across multiple network‐based parcellations for the commonly defined functional networks across atlases, using the whole‐brain ABIDE data rather than the summarized ROI‐level data, may help disentangle variability due to network specification from that due to parcellation boundaries.

## 5 Conclusion

This study investigates the downstream effects of preprocessing pipeline, band‐pass filtering, and brain parcellation on functional connectivity estimates within seven common functional networks, using large‐scale, multi‐site fMRI data. We have found that pipeline and brain parcellation choices significantly influence connectivity estimates, both additively and through interaction effects, while band‐pass filtering shows a limited effect. Our findings highlight the importance of clear articulation and justification of preprocessing methods, as researcher degrees of freedom most definitely apply to the preprocessing of fMRI data and affect the reproducibility and replicability of results. While there are some unique barriers to overcome within fMRI research to fully embrace open science practices, reproducible and replicable research practices are essential for advancing the field and ensuring reliable scientific progress.

## Funding

The authors have nothing to report.

## Supporting information


**Figure S1:** Dendrogram from performing agglomerative hierarchical clustering with average link‐ age on the mean Fisher's *z*‐transformed within‐network values by processing combination. The dendrogram is cut off at a height of 1.5 to form the five clusters shown within the boxes. These clusters correspond to the clusters used in Figure 1C.
**Figure S2:** UMAP results for the projection of only the vectorized within‐network Fisher's z‐transformed values after subtracting out the subject‐ and site‐specific random effects. (A) and (B) UMAP results for pipeline, and faceted by pipeline. For this subset of the overall network, the projection shows up in different parts of the space, but the patterns remain the same as discussed in the main paper. (C) and (D) UMAP results for atlas, and faceted by atlas. The atlas effects within‐network do show up differently than for the full network discussed in the main paper. In panel (C), the cluster on the far right has a mixture of points from the AAL, EZ, HO, and TT atlases. There is a similar cluster in the full network results, but it does not include the TT atlas, and even HO is separated in space from AAL and EZ. Additionally, the cluster in panel (C) corresponds with the NIAK pipeline, shown in panel (A), which matches with one of the five hierarchical clusters in Figure S1. The other two NIAK clusters in panel (A) correspond with the CC200 and CC400 atlases in one group, and the DOS160 atlas in the other in panel (C), which again match with other clusters identified in Figure S1. These results help clearly identify the interaction effect between pipeline and atlas for the within‐network connections.
**Figure S3:** UMAP results for the projection of only the vectorized between‐network Fisher's z‐transformed values after subtracting out the subject‐ and site‐specific random effects. (A) and (B) UMAP results for pipeline, and faceted by pipeline. (C) and (D) UMAP results for atlas, and faceted by atlas. For this subset of the overall network, the projection shows up in different parts of the space, but the patterns remain the same as discussed in the main paper.
**Figure S4:** UMAP results for the projection of the vectorized full network after ComBat harmonization (Yu et al. 2018) for the site‐specific effect and then subtracting out only the subject‐specific random effect. (A) and (B) UMAP results for pipeline, and faceted by pipeline. (C) and (D) UMAP results for atlas, and faceted by atlas. The projection shows up in different parts of the space, but the same patterns hold as when we subtract out the subject‐ and site‐specific random effects instead of using ComBat harmonization.
**Figure S5:** Bootstrap density plots are shown for all (42)=6 pipeline pairwise comparisons for the difference in Frobenius norm. Each subplot contains 100 density curves (black lines) from a sample size of 282 subjects, one for each bootstrapped dataset. The red line in each subplot is the interpolated average density curve for each pipeline pairwise comparison. When the baseline bootstrapping procedure is performed correctly and over a sufficient number of replicates, then all six of the red curves should be nearly identical, as the baseline bootstrap curves are only resampling the edgewise LMM errors, and the fixed effects from the models are removed. These interpolated red curves represent the bootstrap baseline used in the Frobenius norm comparison in the main paper. The same procedure is done for the (72)=21 atlas pairwise comparisons, and achieves the same results, validating the baseline bootstrapping approach for the Frobenius norm of the difference in FC networks for each subject.
**Figure S6:** Bootstrap density plots are shown for all (42)=6 pipeline pairwise comparisons for the portrait divergence. Each subplot contains 100 density curves (black lines) from a sample size of 282 subjects, one for each bootstrapped dataset. The red line in each subplot is the interpolated average density curve for each pipeline pairwise comparison. When the baseline bootstrapping procedure is performed correctly and over a sufficient number of replicates, then all six of the red curves should be nearly identical, as the baseline bootstrap curves are only resampling the edgewise LMM errors, and the fixed effects from the models are removed. These interpolated red curves represent the bootstrap baseline used in the portrait divergence comparison in the main paper. The same procedure is done for the (72)=21 atlas pairwise comparisons, and achieves the same results, validating the baseline bootstrapping approach for the portrait divergence between FC networks for each subject.
**Table S1:** Mean partial sums of squares by network and between network for the edgewise additive effects models.
**Table S2:** Mean partial sums of squares between each pair of networks for the edgewise full models.
**Table S3:** Mean partial sums of squares by network and between networks for the edgewise full models, including MR scanner brand.

## Data Availability

The data that support the findings of this study are openly available from the Autism Brain Imaging Data Exchange at http://preprocessed‐connectomes‐project.org/abide/index.html. All code to reproduce the results can be accessed using our Github repository, https://github.com/kaitlyn‐fales/FC‐Network‐Replicability‐Effects.
