# Supplementary material for: Replicability of Functional Brain Networks: A Study Through the Lens of Seven Resting‐State Networks
Source: Hum Brain Mapp. 2026 Jun 8;47(8):e70559. doi: 10.1002/hbm.70559 (PMC13247136; doi:10.1002/hbm.70559)
Supplement: Supplementary file 1 — Figure S1: Dendrogram from performing agglomerative hierarchical clustering with average link‐ age on the mean Fisher's z‐transformed within‐network values by processing combination. The dendrogram is cut off at a height of 1.5 to form the five clusters shown within the boxes. These clusters correspond to the clusters used in Figure 1C. Figure S2: UMAP results for the projection of only the vectorized within‐network Fisher's z‐transformed values after subtracting out the subject‐ and site‐specific random effects. (A) and (B) UMAP results for pipeline, and faceted by pipeline. For this subset of the overall network, the projection shows up in different parts of the space, but the patterns remain the same as discussed in the main paper. (C) and (D) UMAP results for atlas, and faceted by atlas. The atlas effects within‐network do show up differently than for the full network discussed in the main paper. In panel (C), the cluster on the far right has a mixture of points from the AAL, EZ, HO, and TT atlases. There is a similar cluster in the full network results, but it does not include the TT atlas, and even HO is separated in space from AAL and EZ. Additionally, the cluster in panel (C) corresponds with the NIAK pipeline, shown in panel (A), which matches with one of the five hierarchical clusters in Figure S1. The other two NIAK clusters in panel (A) correspond with the CC200 and CC400 atlases in one group, and the DOS160 atlas in the other in panel (C), which again match with other clusters identified in Figure S1. These results help clearly identify the interaction effect between pipeline and atlas for the within‐network connections. Figure S3: UMAP results for the projection of only the vectorized between‐network Fisher's z‐transformed values after subtracting out the subject‐ and site‐specific random effects. (A) and (B) UMAP results for pipeline, and faceted by pipeline. (C) and (D) UMAP results for atlas, and faceted by atlas. For this subset of the overall netw [file HBM-47-e70559-s001.pdf]

Supplementary Materials

S1 Quality Control Protocol

As our primary goal is to study the replicability of functional networks, we must eliminate, or at least mitigate as many sources of additional variation as possible prior to the analysis. With rs-fMRI analyses, we also should be especially careful in regards to the amount of motion allowed from each subject. We leverage the automated and manual quality assessment (QA) protocol provided by the ABIDE (2013) developers to control for additional sources of variation at the subject level that may have otherwise affected the analysis. Recall, the original sample size for the typical controls in the ABIDE I dataset is 573 subjects.

First, two subjects have completely empty data files, reducing the sample size to 571. Second, we only analyze subjects who self-identify as right-handed (363) or initially left- but switched to right-handed (1), for a sample size of 364. This is the largest reduction in sample size, due to 167 missing or blank handedness observations in addition to a small number of left-handed or ambidextrous subjects. It is well-known that differences in handedness affect functional connectivity estimates (Pool et al., 2015; Tejavibulya et al., 2022, 2025; Tomasi & Volkow, 2024), so it is important that we eliminate this source of variation. Third, to address motion, the automated QA protocol from ABIDE reports the mean framewise displacement (FD) for each subject, as well as the percentage of volumes with FD greater than 0.2 mm. We restrict our sample to subjects with a mean FD less than or equal to 0.2 mm, and a percentage of volumes with FD greater than 0.2 mm less than or equal to 25%. The former controls for consistent motion throughout the scan, which reduces the sample to 318, while the latter controls for possible motion spikes, bringing the sample size to 307 subjects.

Fourth, the manual QA protocol from ABIDE uses three independent raters to assess the quality of the functional images for every subject. The first rater provides either an “OK” or “fail” rating, and the second and third raters provide an “OK”, “maybe”, or “fail” rating. We include only the subjects who received an “OK” from the first rater, and either an “OK” or a “maybe” from the second and third raters, which results in a sample of 283 subjects. From the original sample of 573 typical controls, there are seventeen unique neuroimaging sites, each with several subjects, but after specifying the inclusion criteria described above, only one subject from Carnegie Mellon University (CMU) is included. As the site variation is confounded with the individual variation for this subject, we drop this subject from the analysis.

After the QA protocol, we inspect the data from the remaining subjects, and another 14 have missing values for ROIs where the BOLD signals contain only zeroes. The missingness

does not occur across all processing combinations, but for those it does, we discard the data. This results in a sample size of $n = 282$, but some processing combinations contain data from as few as 268 subjects, making the dataset slightly imbalanced across combinations. For more information on the automated and manual QA protocol, we refer the reader to the ABIDE website (http://preprocessed-connectomes-project.org/abide/quality_assessment.html).

S2 Preprocessing Pipeline Differences

The ABIDE developers preprocess the data using four distinct pipelines, using the default parameters and settings of the original pipeline developers (ABIDE, 2013). Here, we provide additional details regarding the implementation of each of the four pipelines used. All information presented below is drawn from the pipeline documentation on the ABIDE website (http://preprocessed-connectomes-project.org/abide/Pipelines.html). We focus on the functional image preprocessing and refer the reader to the ABIDE documentation for further questions regarding anatomical image preprocessing.

In the descriptions below, we include the appropriate software or tool as explicitly mentioned in the ABIDE documentation whenever available, and do not infer the software (or tool) used for steps in which it is unclear. Some methods used may also be unique to the specific pipeline. As these pipelines may have been updated since their implementation for the ABIDE I data, we do not mention any other software or tools that are only reported in the most updated documentation for each pipeline, as they may not be accurate for the steps performed at the time of the ABIDE I data preprocessing.

Before describing the details of the individual pipelines, we note that four strategies are used for each pipeline, which are all combinations of 0.01-0.1 Hz band-pass filtering (Yes/No) and global signal regression (Yes/No). For the strategies that use global signal regression, the global signal is included as a predictor in the nuisance variable regression step within each pipeline. For the strategies using band-pass filtering, the temporal filtering is applied in the step immediately following nuisance variable regression for all pipelines. As described in the manuscript, we do not consider the strategies that include global signal regression.

S2.1 Connectome Computation System (CCS)

The CCS pipeline (Xing et al., 2022; Xu et al., 2015) first drops the first four volumes of functional images before removing spikes, and performing slice-timing and motion correction, all using the standard AFNI tools (Cox, 1996). The next step includes registering the anatomical brain mask to the functional images using FLIRT in FSL (Jenkinson et al., 2012), and then intersecting this with 3dAutomask in AFNI to get the functional brain mask. A 4D global mean-based procedure is used for intensity normalization, followed by boundary-based registration in Freesurfer (Fischl, 2012). The anatomical segmentation of grey matter, white matter, and cerebrospinal fluid is applied to the functional images using Freesurfer to extract the signals for use in nuisance signal regression. Nuisance correction includes Friston's 24-parameter motion signal (Friston et al., 1996), the mean signal from white matter and cerebrospinal fluid, and linear and quadratic trends. Temporal filtering

is performed using AFNI (if applicable), and finally the functional images are registered to MNI space using FSL.

S2.2 Configurable Pipeline for the Analysis of Connectomes (CPAC)

The CPAC pipeline begins by performing slice-timing correction and then two iterations of motion correction to the average image before skull-stripping for a functional brain mask. All three of these steps use tools from AFNI. Next, 4D global mean intensity normalization is applied. Following that, nuisance signal regression includes Friston's 24-parameter motion model, the top five principal components in the white matter and cerebrospinal fluid, plus linear and quadratic trends. Then, band-pass filtering is performed (if applicable). The functional images are registered to anatomical space using a linear transformation followed by a white matter boundary based registration using FLIRT in FSL plus the white matter tissue segmentation from FSL FAST. Last, the functional images are registered to MNI space using ANTs (Tustison et al., 2021).

S2.3 Data Processing Assistant for Resting-State fMRI (DPARSF)

The DPARSF pipeline, like CCS, drops the first four volumes of functional images at the beginning of preprocessing. After that, slice-timing correction is performed before applying a six-parameter rigid body transformation for motion realignment of the functional images. The mean functional image is then co-registered to the structural images. For nuisance signal correction, Friston's 24-parameter motion model, the mean white matter and cerebrospinal fluid signals, as well linear and quadratic trends are used as regressors. Next, temporal filtering is performed (if applicable). The functional images are registered into MNI space using the transformation information acquired from DARTEL (Ashburner, 2007) for the structural images. Finally, the images are smoothed using a 6 mm Gaussian kernel.

S2.4 Neuroimaging Analysis Kit (NIAK)

The NIAK pipeline (release 0.7.1) does not drop any functional volumes or apply any form of slice-timing correction. For intensity normalization, a non-uniformity correction using the median functional volume is applied to all volumes in the run using the N3 method, which estimates and removes a smooth intensity bias field (Sled et al., 1998). Then, a rigid-body motion realignment procedure is performed using the median volume as the target. The functional volumes are resampled into MNI space using CIVET (Zijdenbos et al., 2002), and then the scrubbing method from Power et al. (2012) is used to remove volumes with

excessive motion, defined as volumes with a framewise displacement greater than 0.5 mm. Next, nuisance signal regression includes the first principal component of the six head motion parameters and their squares, the mean white matter and cerebrospinal fluid signals, and a discrete cosine basis with a 0.01 Hz high-pass cut-off for low-frequency drifts. Based on the description from ABIDE, temporal filtering is then performed (if applicable), but this is not explicitly mentioned in the NIAK portion of the documentation. The functional volumes are then spatially smoothed using a 6 mm Gaussian kernel.

S3 Tables and Figures

Here we present several supplementary tables and figures pertaining to the hierarchical clustering results, the linear mixed effects model results for the additive effects models and between-network full models, UMAP results for within-network, between-network, as well as the results post-ComBat (Yu et al., 2018) harmonization. The baseline bootstrap density plots for the pipeline pairwise comparisons for both the Frobenius norm and portrait divergence are also shown and discussed.

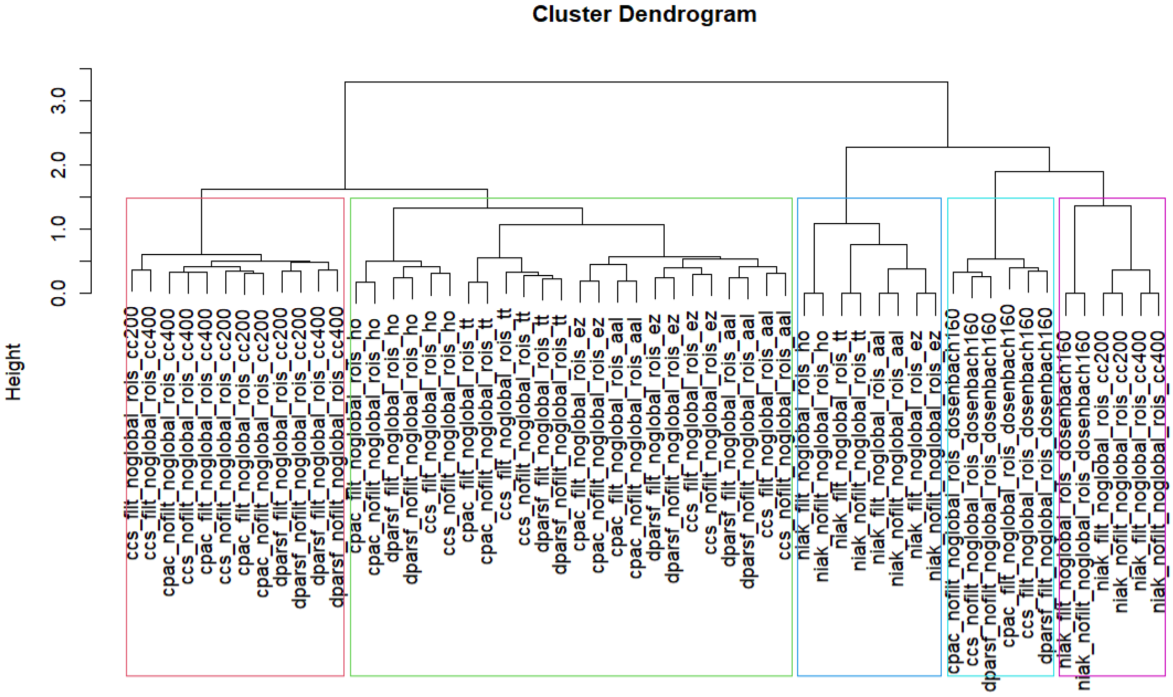

Figure S1: Dendrogram from performing agglomerative hierarchical clustering with average linkage on the mean Fisher's z-transformed within-network values by processing combination. The dendrogram is cut off at a height of 1.5 to form the five clusters shown within the boxes. These clusters correspond to the clusters used in Figure 1 Panel C of the main text.

Table S1: Mean partial sums of squares by network and between network for the edgewise additive effects models.

	DMN	SMN	VN	SN	DAN	FPN	LN	BN
**Pipeline**	331.57	750.88	354.10	378.54	300.04	700.75	370.77	137.70
**Filter**	2.90	8.20	1.46	4.28	1.40	3.58	1.85	1.05
**Atlas**	313.92	1681.73	1137.37	226.01	490.48	243.88	404.28	667.41

The mean partial sum of squares by network and for edges between any two networks (BN) results from the edgewise additive effects models. There are main effects for pipeline and atlas, which motivates further exploration of these effects including interaction terms in the full models.

Table S2: Mean partial sums of squares between each pair of networks for the edgewise full models.

	BN Average	DAN & DMN	DAN & SN	DAN & SMN	DAN & VN
**Pipeline**	137.70	102.58	158.14	113.09	143.58
**Filter**	1.05	1.22	2.28	0.66	1.11
**Atlas**	667.53	753.16	1177.07	713.53	304.21
**Pipeline:Filter**	0.66	0.67	1.10	0.49	0.54
**Pipeline:Atlas**	26.80	46.32	32.38	29.57	19.11
**Filter:Atlas**	0.34	0.49	0.51	1.01	0.25
**Pipeline:Filter:Atlas**	0.15	0.23	0.22	0.37	0.14
	**FPN & DMN**	**FPN & DAN**	**FPN & SN**	**FPN & SMN**	**FPN & VN**
**Pipeline**	194.10	132.09	218.79	45.46	107.53
**Filter**	0.48	0.81	1.56	0.68	0.07
**Atlas**	592.03	1578.51	1179.97	473.58	343.57
**Pipeline:Filter**	0.45	0.53	0.85	0.58	0.32
**Pipeline:Atlas**	31.78	64.60	33.88	19.80	15.96
**Filter:Atlas**	0.23	0.42	0.56	0.22	0.10
**Pipeline:Filter:Atlas**	0.12	0.19	0.23	0.09	0.05
	**LN & DMN**	**LN & DAN**	**LN & FPN**	**LN & SN**	**LN & SMN**
**Pipeline**	144.33	147.35	245.71	209.67	92.47
**Filter**	0.29	1.97	2.21	1.45	1.20
**Atlas**	562.20	532.51	497.99	660.96	434.97
**Pipeline:Filter**	0.38	1.02	1.13	0.84	0.74
**Pipeline:Atlas**	20.63	43.86	27.62	23.92	22.22
**Filter:Atlas**	0.18	0.41	0.13	0.43	0.32
**Pipeline:Filter:Atlas**	0.10	0.18	0.08	0.19	0.14
	**LN & VN**	**SN & DMN**	**SN & SMN**	**SN & VN**	**SMN & DMN**
**Pipeline**	88.93	97.96	152.76	112.35	43.13
**Filter**	0.20	0.77	1.84	0.14	1.01
**Atlas**	302.50	646.45	510.24	205.62	556.70
**Pipeline:Filter**	0.35	0.62	1.00	0.37	0.57
**Pipeline:Atlas**	10.09	29.58	21.89	10.46	14.88
**Filter:Atlas**	0.15	0.46	0.28	0.15	0.21
**Pipeline:Filter:Atlas**	0.07	0.19	0.12	0.07	0.10
	**VN & DMN**	**VN & SMN**			
**Pipeline**	67.11	120.10			
**Filter**	0.10	0.73			
**Atlas**	1159.62	257.06			
**Pipeline:Filter**	0.35	0.41			
**Pipeline:Atlas**	30.08	8.78			
**Filter:Atlas**	0.26	0.11			
**Pipeline:Filter:Atlas**	0.11	0.05			

The mean partial sums of squares between all pairs of networks from the Type III ANOVA are shown for the edgewise full models. The main effects of pipeline and atlas, and the interaction of pipeline and atlas are notable. In comparison to the within-network models, the pipeline effect is not as strong, and neither is the interaction effect. There are no discernible patterns between pairs of networks, so we summarize the between network results by taking the mean sum of squares across all edges between networks (BN Average) to report in the main text.

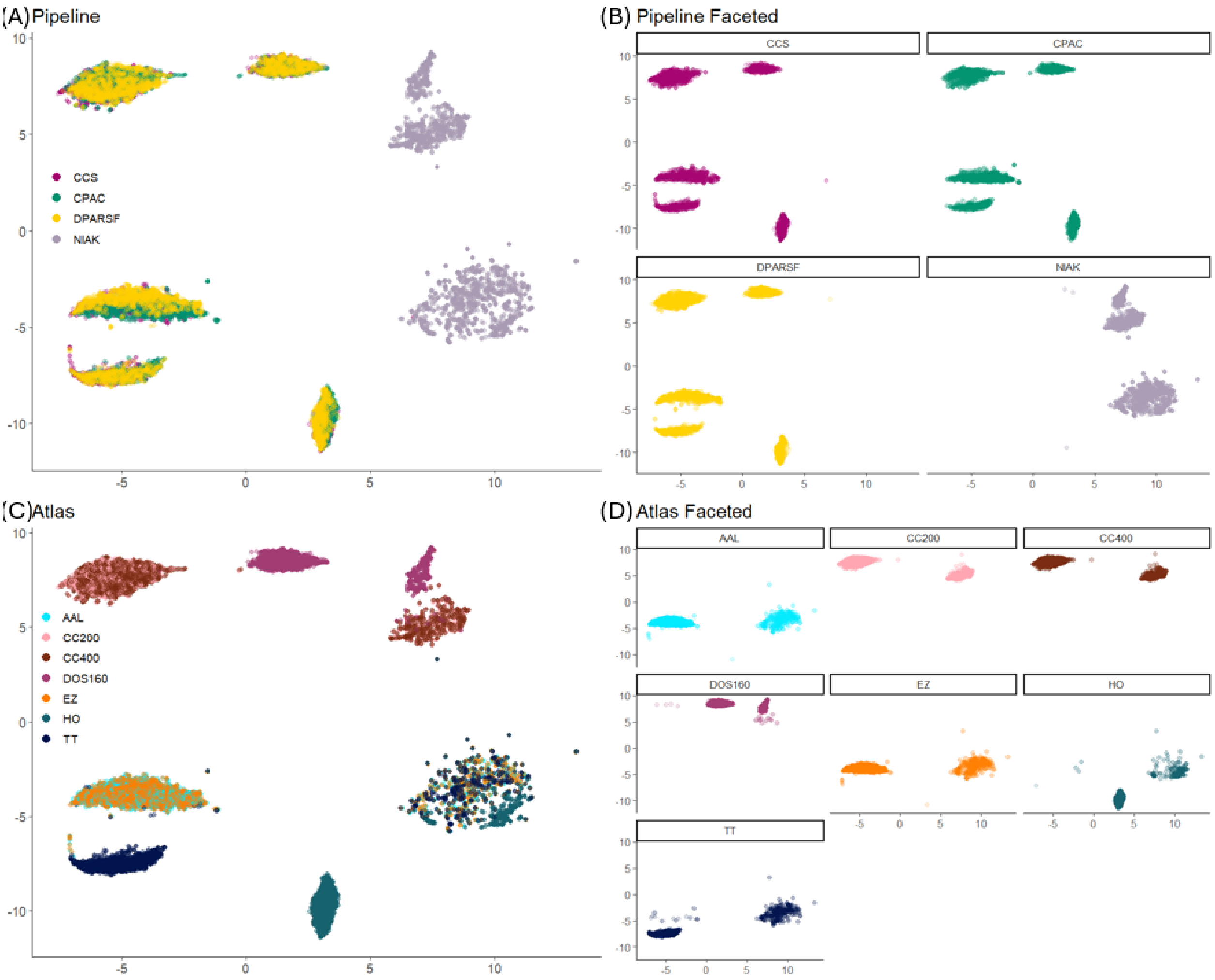

Figure S2: UMAP results for the projection of only the vectorized *within-network* Fisher's z-transformed values after subtracting out the subject- and site-specific random effects. (A)-(B) UMAP results for pipeline, and faceted by pipeline. For this subset of the overall network, the projection shows up in different parts of the space, but the patterns remain the same as discussed in the main text. (C)-(D) UMAP results for atlas, and faceted by atlas. The atlas effects *within-network* do show up differently than for the full network discussed in the main text. In panel (C), the cluster on the far right has a mixture of points from the AAL, EZ, HO, and TT atlases. There is a similar cluster in the full network results, but it does not include the TT atlas, and even HO is separated in space from AAL and EZ. Additionally, the cluster in panel (C) corresponds with the NIAK pipeline, shown in panel (A), which matches with one of the five hierarachical clusters in Figure S1. The other two NIAK clusters in panel (A) correspond with the CC200 and CC400 atlases in one group, and the DOS160 atlas in the other in panel (C), which again match with other clusters identified in Figure S1. These results help clearly identify the interaction effect between pipeline and atlas for the *within-network* connections.

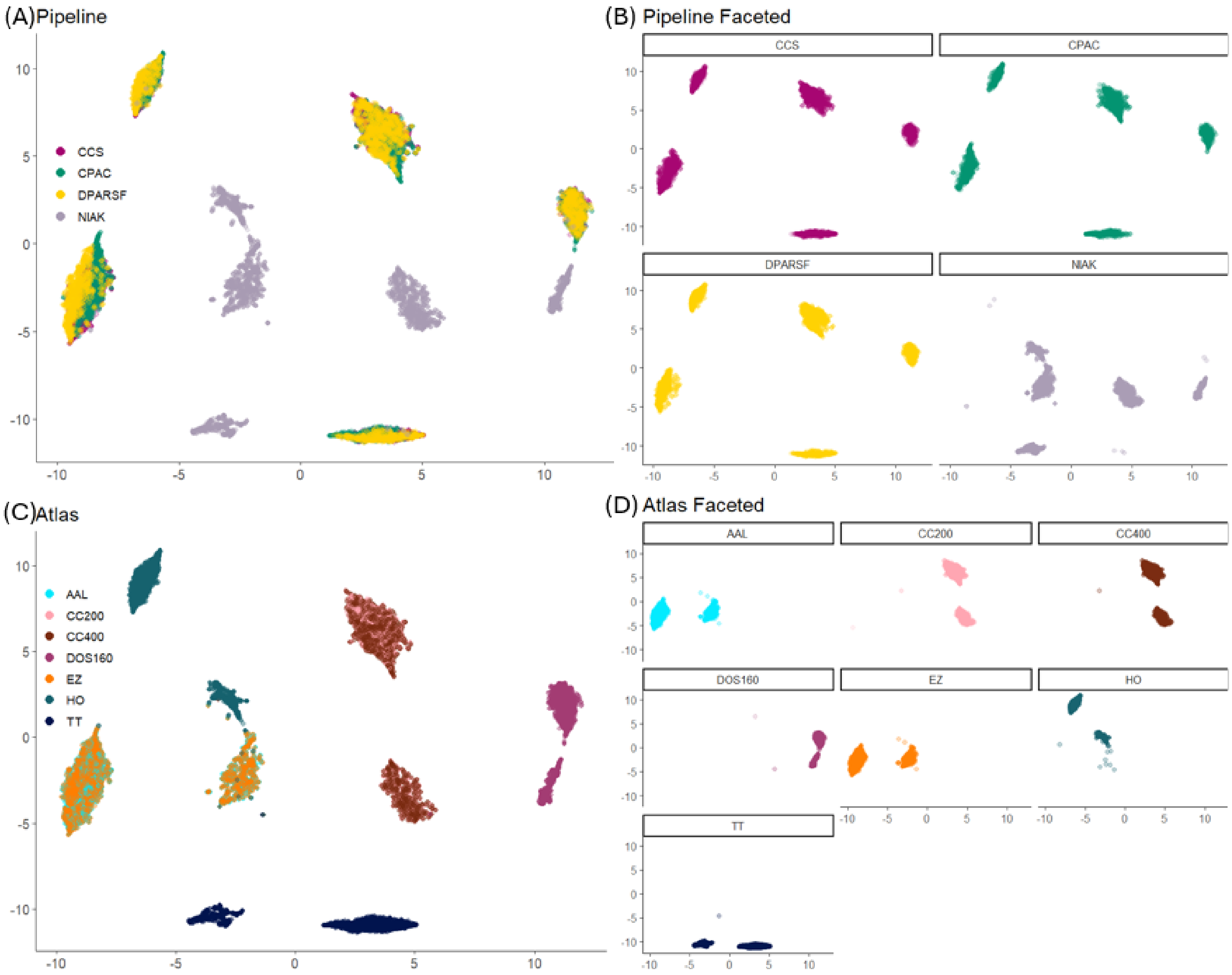

Figure S3: UMAP results for the projection of only the vectorized *between-network* Fisher's z-transformed values after subtracting out the subject- and site-specific random effects. (A)-(B) UMAP results for pipeline, and faceted by pipeline. (C)-(D) UMAP results for atlas, and faceted by atlas. For this subset of the overall network, the projection shows up in different parts of the space, but the patterns remain the same as discussed in the main text.

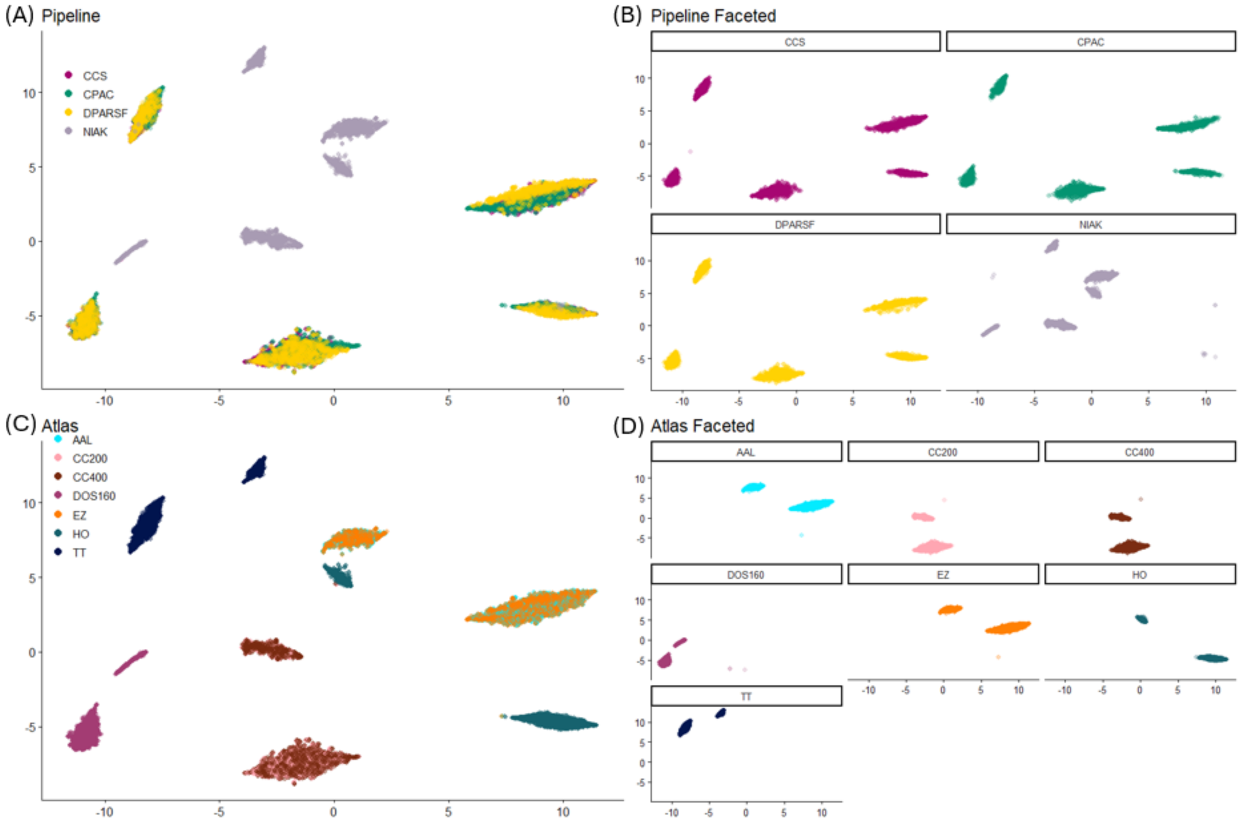

Figure S4: UMAP results for the projection of the vectorized full network after ComBat harmonization (Yu et al., 2018) for the site-specific effect and then subtracting out only the subject-specific random effect. (A)-(B) UMAP results for pipeline, and faceted by pipeline. (C)-(D) UMAP results for atlas, and faceted by atlas. The projection shows up in different parts of the space, but the same patterns hold as when we subtract out the subject- and site-specific random effects instead of using ComBat harmonization.

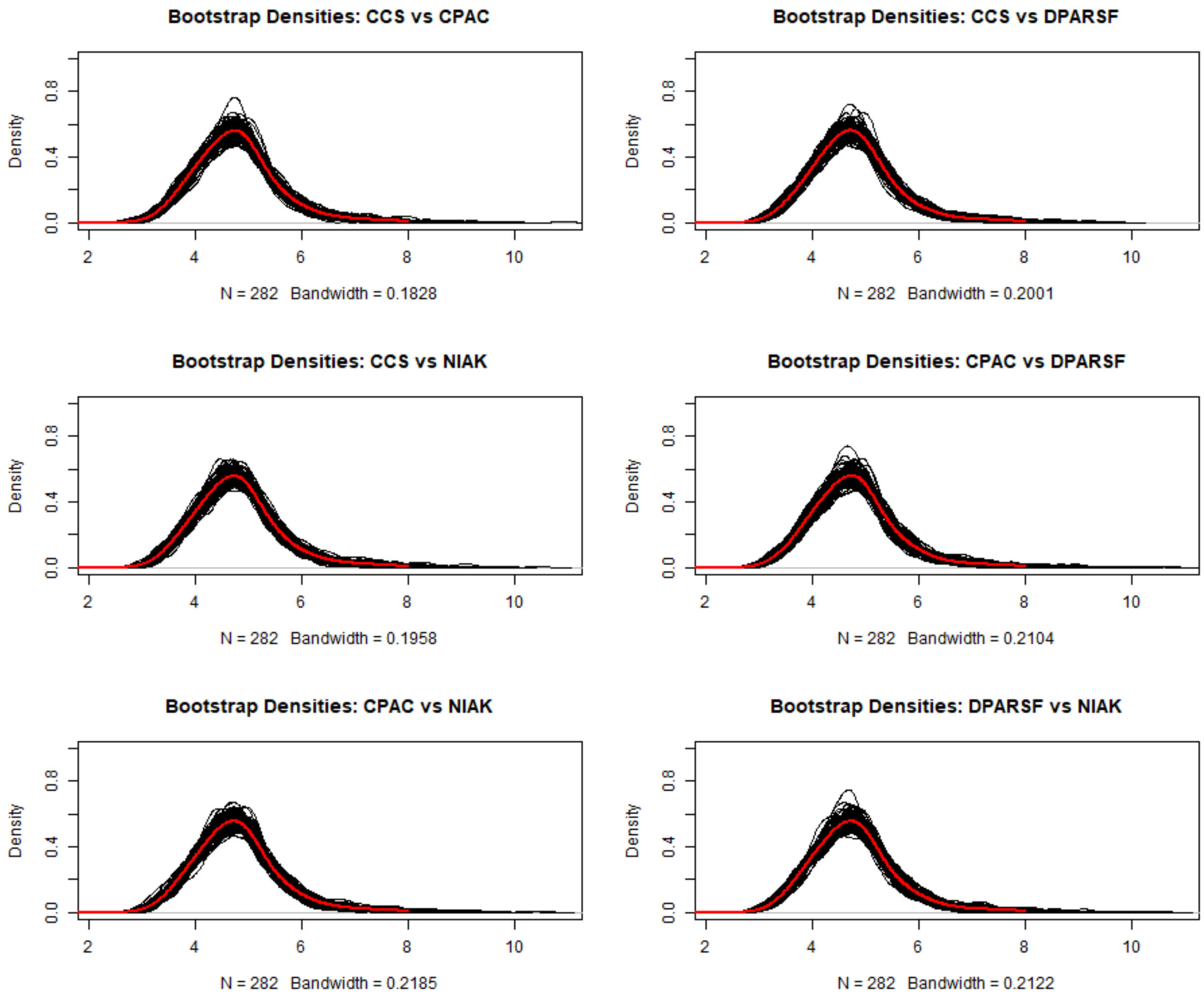

Figure S5: Bootstrap density plots are shown for all $\binom{4}{2} = 6$ pipeline pairwise comparisons for the difference in Frobenius norm. Each subplot contains 100 density curves (black lines) from a sample size of 282 subjects, one for each bootstrapped dataset. The red line in each subplot is the interpolated average density curve for each pipeline pairwise comparison. When the baseline bootstrapping procedure is performed correctly and over a sufficient number of replicates, then all six of the red curves should be nearly identical, as the baseline bootstrap curves are only resampling the edgewise LMM errors, and the fixed effects from the models are removed. These interpolated red curves represent the bootstrap baseline used in the Frobenius norm comparison in the main text. The same procedure is done for the $\binom{7}{2} = 21$ atlas pairwise comparisons, and achieves the same results, validating the baseline bootstrapping approach for the Frobenius norm of the difference in FC networks for each subject.

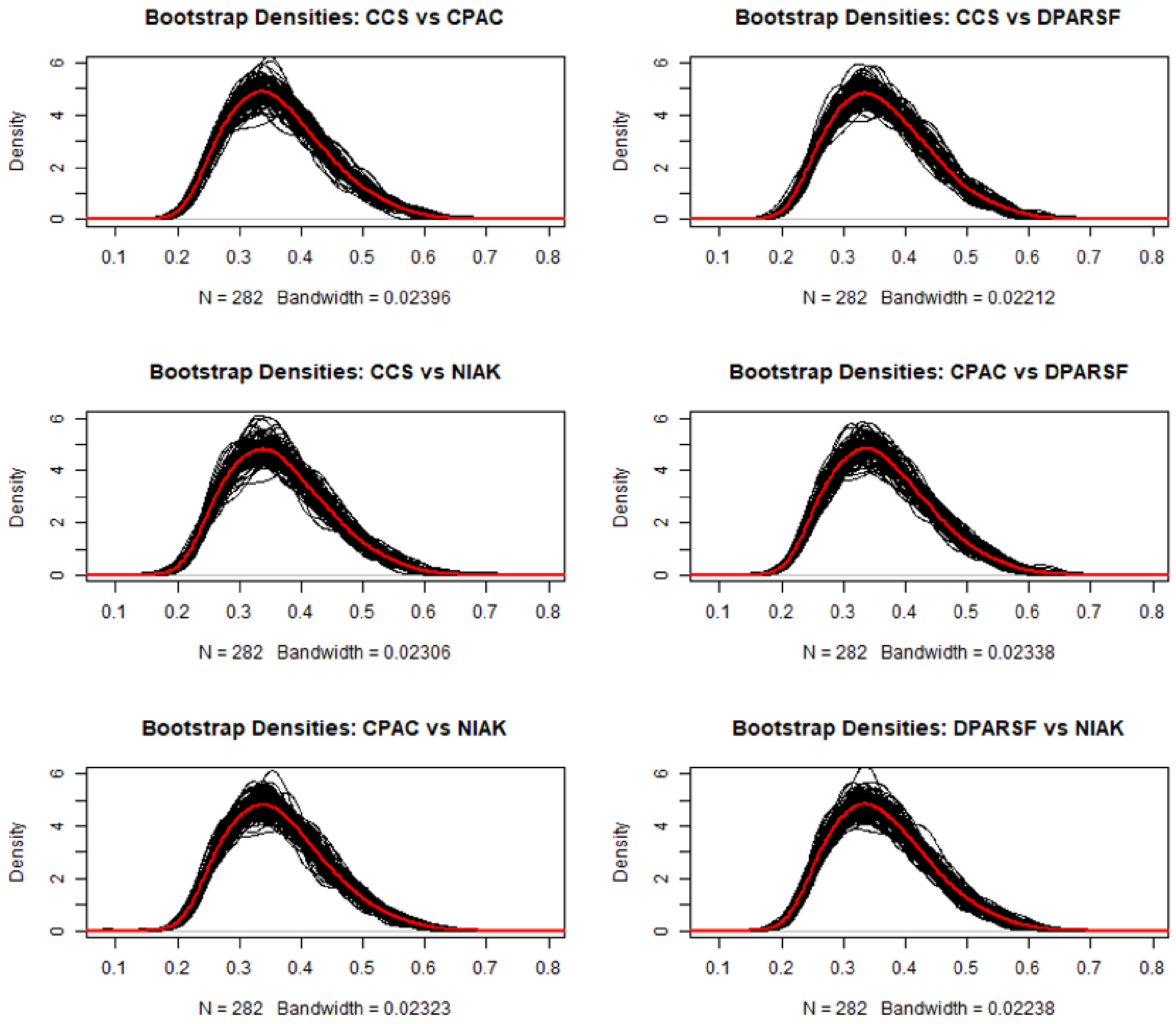

Figure S6: Bootstrap density plots are shown for all $\binom{4}{2} = 6$ pipeline pairwise comparisons for the portrait divergence. Each subplot contains 100 density curves (black lines) from a sample size of 282 subjects, one for each bootstrapped dataset. The red line in each subplot is the interpolated average density curve for each pipeline pairwise comparison. When the baseline bootstrapping procedure is performed correctly and over a sufficient number of replicates, then all six of the red curves should be nearly identical, as the baseline bootstrap curves are only resampling the edgewise LMM errors, and the fixed effects from the models are removed. These interpolated red curves represent the bootstrap baseline used in the portrait divergence comparison in the main text. The same procedure is done for the $\binom{7}{2} = 21$ atlas pairwise comparisons, and achieves the same results, validating the baseline bootstrapping approach for the portrait divergence between FC networks for each subject.

S4 MR Scanner Brand Modeling and Results

While the primary interest in the current study is the effect of pipeline, band-pass filtering, and brain parcellation on FC network replicability, a secondary interest is the potential implications of also considering MR scanner brand. Across the sixteen unique neuroimaging sites, there are three scanner brands: Siemens, Phillips, and GE. To study the effect of MR scanner brand, we construct a third linear mixed effects model that is identical to the full model for each edge, but also include the additive fixed effect of MR scanner brand, $\delta_o^{(e)}$ for each network edge e. The model is given by, for each subject i and edge e,

$$z_{ijk\ell}^{(e)} = (\mu^{(e)} + \zeta_m^{(e)} + \eta_{i(m)}^{(e)}) + \alpha_j^{(e)} + \beta_k^{(e)} + \gamma_\ell^{(e)} + (\alpha\beta)_{jk}^{(e)} + (\alpha\gamma)_{j\ell}^{(e)} + (\beta\gamma)_{k\ell}^{(e)} + (\alpha\beta\gamma)_{jk\ell}^{(e)} + \delta_o^{(e)} + \epsilon_{ijk\ell}^{(e)},$$

where $\zeta_m^{(e)} \sim \mathrm{N}(0, (\sigma_\zeta^{(e)})^2)$ are site-specific random effects that are independent across sites m and network edges e, $\eta_{i(m)}^{(e)} \sim \mathrm{N}(0, (\sigma_\eta^{(e)})^2)$ are subject-specific effects nested within sites, which are independent across sites m, subjects i, and network edges e, and $\epsilon_{ijk\ell}^{(e)} \overset{\text{iid}}{\sim} \mathrm{N}(0, (\sigma^{(e)})^2)$ are the errors. Even when two sites use the same scanner brand and model, there are differences in the scanning parameters being chosen (e.g., TE, flip angle, etc.). As such, the scanner brand effect $\delta_o^{(e)}$ is not truly the isolated effect of the brand, but rather the additional variation that can be attributed to brand beyond neuroimaging site variation, as they are confounded.

From the edgewise linear mixed models including MR scanner brand we do not observe

Table S3: Mean partial sums of squares by network and between networks for the edgewise full models, including MR scanner brand.

	DMN	SMN	VN	SN	DAN	FPN	LN	BN
**Pipeline**	323.17	733.17	344.95	364.90	289.48	679.26	360.63	132.59
**Filter**	2.63	7.44	1.18	3.84	1.09	3.13	1.59	0.93
**Atlas**	302.40	1621.35	1098.63	215.39	469.99	235.88	387.99	643.70
**MR Scanner Brand**	0.13	0.32	0.08	0.10	0.35	0.19	0.10	0.10
**Pipeline:Filter**	1.05	2.76	0.63	1.58	0.57	1.30	0.96	0.60
**Pipeline:Atlas**	44.46	61.42	17.71	41.01	55.97	20.48	23.52	25.82
**Filter:Atlas**	0.62	0.84	0.69	0.85	2.07	0.76	0.37	0.32
**Pipeline:Filter:Atlas**	0.26	0.33	0.26	0.33	0.76	0.30	0.18	0.14

The average partial sums of squares within and between networks from the Type III ANOVA are shown for the edgewise full models when including the additive effect of MR scanner brand (highlighted), of which there are three levels: Siemens, Phillips, and GE. The other sums of squares remain largely the same, and brand overall accounts for little to no variation within the FC network. However, for the SMN and DAN, the sum of squares contribution is larger compared to the other networks.

any effects. The results from the models are shown in Table S3, where we observe that the scanner brand partial sum of squares is nearly zero in all cases. Overall, little to no variation can be attributed to the scanner brands themselves, which suggests that the brands do not contribute to variation nearly as much as the scanning sites.

References

ABIDE. (2013). Abide preprocessed. http://preprocessed-connectomes-project.org/abide/index.html

Ashburner, J. (2007). A fast diffeomorphic image registration algorithm. *NeuroImage*, *38*, 95–113.

Cox, R. (1996). AFNI: Software for analysis and visualization of functional magnetic resonance neuroimages. *Computers and Biomedical Research, an International Journal*, *29*(3). https://doi.org/10.1006/cbmr.1996.0014

Fischl, B. (2012). FreeSurfer. *NeuroImage*, *62*(2), 774–781. https://doi.org/10.1016/j.neuroimage.2012.01.021

Friston, K., Williams, S., Howard, R., Frackowiak, R., & Turner, R. (1996). Movement-related effects in fMRI time-series. *Magnetic resonance in medicine*, *35*(3), 346–355. https://doi.org/10.1002/mrm.1910350312

Jenkinson, M., Beckmann, C., Behrens, T., Woolrich, M., & Smith, S. (2012). FSL. *NeuroImage*, *62*(2), 782–790. https://doi.org/10.1016/j.neuroimage.2011.09.015

Pool, E.-M., Rehme, A., Eickhoff, S., Fink, G., & Grefkes, C. (2015). Functional resting-state connectivity of the human motor network: Differences between right- and left-handers. *NeuroImage*, *109*, 298–306. https://doi.org/https://doi.org/10.1016/j.neuroimage.2015.01.034

Power, J., Barnes, K., Snyder, A., Schlaggar, B., & Petersen, S. (2012). Spurious but systematic correlations in functional connectivity mri networks arise from subject motion. *NeuroImage*, *59*(3), 2142–2154. https://doi.org/10.1016/j.neuroimage.2011.10.018

Sled, J., Zijdenbos, A., & Evans, A. (1998). A nonparametric method for automatic correction of intensity nonuniformity in mri data. *IEEE Transactions on Medical Imaging*, *17*, 87–97. https://doi.org/10.1109/42.668698

Tejavibulya, L., Horien, C., Fredricks, C., Ficek-Tani, B., Westwater, M., & Scheinost, D. (2025). Brain handedness associations depend on how and when handedness is measured. *Scientific Reports*, *15*(9674). https://doi.org/10.1038/s41598-025-94036-8

Tejavibulya, L., Peterson, H., Greene, A., Gao, S., Rolison, M., Noble, S., & Scheinost, D. (2022). Large-scale differences in functional organization of left- and right-handed individuals using whole-brain, data-driven analysis of connectivity. *NeuroImage*, *252*(119040). https://doi.org/10.1016/j.neuroimage.2022.119040

Tomasi, D., & Volkow, N. (2024). Associations between handedness and brain functional connectivity patterns in children. *Nature Communications*, *15*(2355). https://doi.org/10.1038/s41467-024-46690-1

Tustison, N., Cook, P., Holbrook, A., Johnson, H., Muschelli, J., Devenyi, G., Duda, J., Das, S., Cullen, N., Gillen, D., Yassa, M., Stone, J., Gee, J., & Avants, B. (2021). The ANTsX ecosystem for quantitative biological and medical imaging. *Scientific Reports*, *11*(9068). https://doi.org/10.1038/s41598-021-87564-6

Xing, X.-X., Xu, T., Jiang, C., Wang, Y.-S., & Zuo, X.-N. (2022). Connectome computation system: 2015-2021 updates. *Science Bulletin*, *67*(5), 448–451. https://doi.org/10.1016/j.scib.2021.11.021

Xu, T., Yang, Z., Xing, X.-X., & Zuo, X.-N. (2015). A connectome computation system for discovery science of brain. *Science Bulletin*, *60*(1), 86–95. https://doi.org/10.1007/s11434-014-0698-3

Yu, M., Linn, K., Cook, P., Phillips, M., McInnis, M., Fava, M., Trivedi, M., Weissman, M., Shinohara, R., & Sheline, Y. (2018). Statistical harmonization corrects site effects in functional connectivity measurements from multi-site fMRI data. *Human Brain Mapping*, *39*(11), 4213–4227. https://doi.org/10.1002/hbm.24241

Zijdenbos, A., Forghani, R., & Evans, A. (2002). Automatic “pipeline”’ analysis of 3-D MRI data for clinical trials: Application to multiple sclerosis. *IEEE Transactions on Medical Imaging*, *21*(10), 1280–1291.
